# Clinical proteomics for prostate cancer: understanding prostate cancer pathology and protein biomarkers for improved disease management

**DOI:** 10.1186/s12014-020-09305-7

**Published:** 2020-11-20

**Authors:** Claire Tonry, Stephen Finn, John Armstrong, Stephen R. Pennington

**Affiliations:** 1 UCD Conway Institute, University College Dublin, Dublin, Ireland; 2 Department of Histopathology and Morbid Anatomy, Trinity Translational Medicine Institute, Trinity College Dublin, Dublin 8, Ireland; 3 St. Luke’s Hospital, Rathgar, Dublin 6, Ireland

**Keywords:** Prostate cancer, Biomarkers, Proteomics, Disease pathology

## Abstract

Following the introduction of routine Prostate Specific Antigen (PSA) screening in the early 1990′s, Prostate Cancer (PCa) is often detected at an early stage. There are also a growing number of treatment options available and so the associated mortality rate is generally low. However, PCa is an extremely complex and heterogenous disease and many patients suffer disease recurrence following initial therapy. Disease recurrence commonly results in metastasis and metastatic PCa has an average survival rate of just 3–5 years. A significant problem in the clinical management of PCa is being able to differentiate between patients who will respond to standard therapies and those who may benefit from more aggressive intervention at an earlier stage. It is also acknowledged that for many men the disease is not life threatenting. Hence, there is a growing desire to identify patients who can be spared the significant side effects associated with PCa treatment until such time (if ever) their disease progresses to the point where treatment is required. To these important clinical needs, current biomarkers and clinical methods for patient stratification and personlised treatment are insufficient. This review provides a comprehensive overview of the complexities of PCa pathology and disease management. In this context it is possible to review current biomarkers and proteomic technologies that will support development of biomarker-driven decision tools to meet current important clinical needs. With such an in-depth understanding of disease pathology, the development of novel clinical biomarkers can proceed in an efficient and effective manner, such that they have a better chance of improving patient outcomes.

## Background

Prostate Cancer (PCa) is the second most common cancer diagnosed in men, and the fifth most common cause of cancer-associated death for males worldwide [1, 2]. Appropriate management of the disease is one of the biggest challenges associated with PCa. The majority of men diagnosed with PCa have indolent disease that can be safely managed without immediate treatment and will likely not be threatening to their natural life expectancy. However, for some the disease will progress and spread (metastasise) to other sites beyond the prostate, at which point the prognosis for patients is much worse. Indeed only 28% of men diagnosed with metastatic PCa survive beyond 5 years [3, 4].

Although PSA remains the gold-standard biomarker for PCa diagnosis and is one of the most widely used blood-based biomarkers in cancer, it contributes significantly to over-treatment of men with PCa [5]. This is a significant issue, as treatment options for PCa are associated with side effects that can have a profound negative impact on quality of life. It is widely acknowledged that new and improved biomarkers are urgently required for management of men diagnosed with PCa and to guide the most appropriate treatment option for individual patients. Ideally such biomarkers would be measurable in a biosample that is available in a minimially invasive manner and amenable for repeat testing.

This review aims to provide a comprehensive oversight of PCa, providing detail on PCa pathology, clinical diagnosis, treatment options, underlying biology and biomarkers associated with the disease. In-depth understanding of PCa pathophysiology will be required in order to identify biomarkers that will likely translate to a clinical test and support individualized treatment options for men with this highly heterogenous and complex disease. In addition, technical advances in the field of proteomics that will support the discovery and validation of effective biomarkers for improved diagnosis and stratification of patients with PCa, will be highlighted.

### Pathology of prostate cancer

**Fig. 1:**
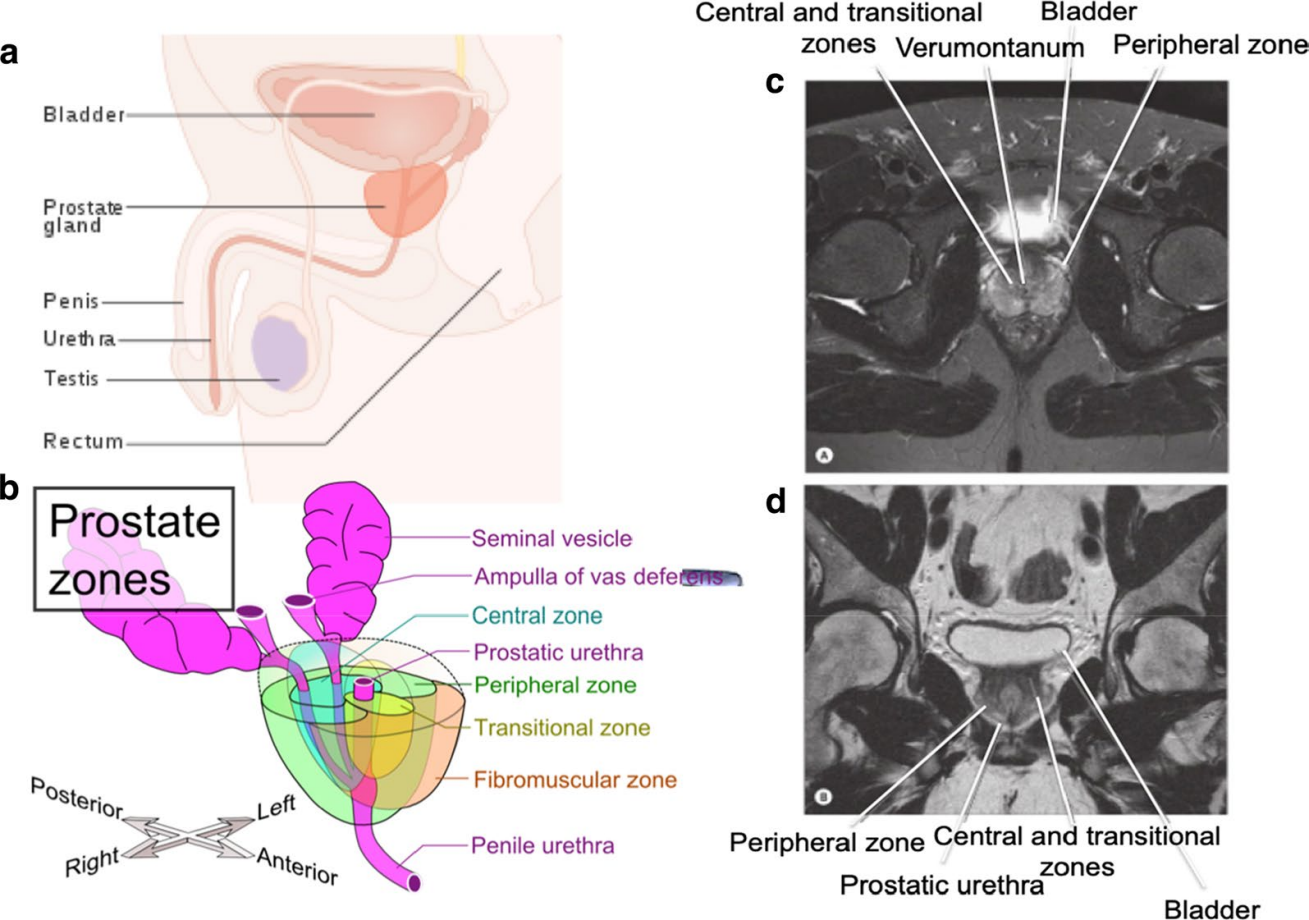
**Position and Zonal Anatomy of the Human Prostate Gland**. Position of the prostate in the male reproductive tract (**a**) and location of the three main anatomical zones of the human prostate as described by McNeal in 1969 (**b**). Zonal regions of the prostate as viewed under magnetic resonance imaging (MRI) are shown in (**c**). Figure is adapted from ‘Perez and Brady’s Principals and Practices of Radiation Oncology’ [275] and ‘Grey’s Anatomy’ [276]

The prostate is the largest accessory gland in the male reproductive system and is located within the lower pelvis between the bladder and the penis [6]. It was once thought that the prostate gland was divided into five anatomical lobes, however, now only three lobes—two anterior and one median—are recognized. McNeal was among the first to describe the three histologically distinct zones of the prostate [7] (Fig. 1). From a clinical perspective, comprehension of the zonal anatomy of the prostate is central to the understanding of both benign and malignant prostatic pathologies, as the zone in which the pathology originates is a defining characteristic of each of the three main prostate diseases. The three main diseases of the prostate are; benign prostate hyperplasia (BPH), prostate carcinoma (PCa) and chronic prostatitis (CP). Chronic prostatitis (CP) is a urological disorder that can encompass many symptomatological patterns, but is formally defined as an inflammation and swelling of the prostate gland [8, 9]. CP affects between 4.5 and 9% of the male population and is the most common urologic diagnosis in men younger than 50 years old [9]. BPH refers to an enlargement of the transitional zone of the prostate and can occur spontaneously in men aged over 50. The increasing size usually occurs as result of decreased testosterone production and increased estrogen production by interstitial cells, which stimulates prostatic growth [10]. The enlarged prostate compresses on the bladder and urethra and so early symptoms of the disease generally include increased urinary frequency, urinary urgency and difficulty in initiating micturition. PCa is associated with similar symptoms to BPH, especially in the early stages. However, in the majority of cases, PCa originates in the peripheral zone of the prostate. PCa is characterized as an adenocarcinoma and shares many similarities with other common epithelial cancers such as breast and colon.

PCa is often a multifocal disease, meaning that numerous tumours can arise within a single patient [11, 12]. In fact, multiple tumours of varying sizes are found in 80% of prostatectomy specimens [13, 14]. Thus, within an individual patient, both interfocal heterogeneity—variations between multiple tumour foci—and intrafocal heterogeneity—variations between tumour cells within the same focus—are common features [15]. The focal origin of PCa is thought to influence its metastatic potential. Indeed, observations made by Guo et al*.* suggest that the genomic profile of tumours differ based on their zonal origin [16]. Tumours that originate in the transition zone of the prostate (~ 20%), are associated with larger tumour volume and higher levels of PSA, but overall more favourable prognosis as compared to tumours that originate in the peripheral zone [16]. Within focal regions, there are areas of both well differentiated, glandular, low-grade tumour tissue as well as poorly differentiated tumour tissue, lacking in glandular structures. Moreover, it is widely accepted that, through genetic mutation or otherwise, prostate tumors contain subpopulations of cells that are (or become) resistant to therapeutic intervention and give rise to cells of metastatic potential [11, 17]. As such, the inter-and intra-focal heterogeneity of PCa complicates diagnosis and treatment of the disease and is a fundamental challenge in the management of this common malignancy. Indeed, it has been observed that, for some proteins, expression variations between patients are equivalent to the extent of variation within a single prostate [18]. This heterogeneity predicates the challenge associated with identifying molecular biomarkers that might be used to inform and direct patient outcomes.

### Prostate cancer incidence and mortality

PCa is the fifth most common cause of cancer-associated death for males worldwide [2]. There are variations in clinical incidence and mortality rates between geographic and ethnic populations that remain poorly understood [15]. For example, the incidence of PCa is much greater in men of African-American ethnicity and the prognosis for African Americans and other minority ethnicities is often worse than for Caucasian men [19, 20]. In contrast, incidence of PCa is significantly lower in Asian men as opposed to Caucasian or African-American ethnicities [10]. Across all races, PCa is considered to be a disease of the elderly, as the likelihood of developing PCa is closely associated with advancing age [21–23]. Indeed, advanced age is the leading risk factor for PCa.

The majority of men diagnosed with PCa will have indolent disease that will not require immediate interventional treatment [24]. However, the prognosis for patients is much worse if the cancer has had a chance to spread. Because of the location of the prostate, metastasis rapidly involves the lymphatic system, lungs, bone marrow, liver or adrenal glands [10]. Despite the advances that have been made in treatment of PCa in recent years, the average survival time for men diagnosed with metastatic PCa is approximately only 2.5 years [25]. In the last 20 years, a man’s lifetime risk of being diagnosed with PCa has increased considerably, which is largely associated with the introduction of PSA screening in the early 1980′s [26, 27]. On the other hand, the percentage of men dying from PCa has decreased, which can in some way be attributed to the fact that nowadays the disease is usually diagnosed and treated at an earlier stage [28].

### Prostate cancer diagnosis and staging

#### The role of prostate specific antigen

**Fig. 2:**
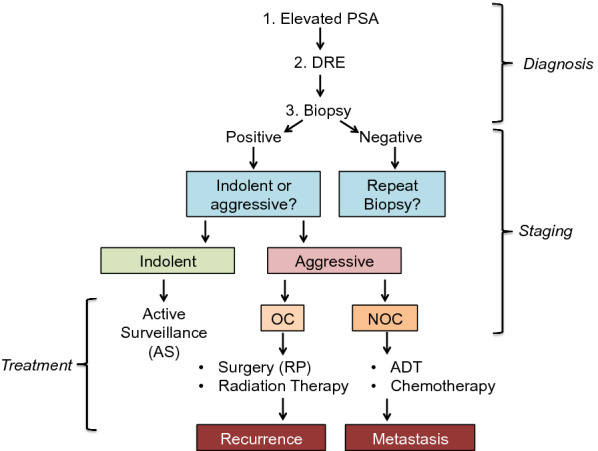
**Clinical Diagnosis, Staging and Treatment Options for Prostate Cancer**. Schematic of the clinical diagnosis, staging and patient stratification for appropriate PCa treatment

Since it was first described as a prostate specific protein, the level of PSA in blood has become the most commonly used molecular marker for screening, diagnosis and management of PCa and indeed is the most widely used screening marker for any cancer [29]. The US food and drug administration (FDA) first approved the sale and use of a PSA test in 1987 and large-scale PSA screening was initiated in the US in 1991. All men ≥ 50 years of age are recommended for PSA screening and it represents the first stage in diagnosis of PCa (Fig. 2). Those with PSA levels ≥ 4.0 ng/mL are recommended for biopsy and those with a positive biopsy are given a diagnosis of PCa [30].

Screening for PSA is sufficiently sensitive to detect many low-risk cancers. However, considering that PCa is a disease which, in many cases may never cause significant harm to a patient, it has also been associated with a large increase in the number of men being over-diagnosed and over-treated for PCa [31, 32]. Observations made from the European Randomized Study of Screening for Prostate Cancer (ERSPC), indicated that PSA screening contributed to a substantial (~ 21%) decrease in PCa mortality after 13 years follow up of non-screened and screened men [33]. However, results from this study did also indicate that, of > 700 men invited for PSA screening, only one PCa-related death was prevented. In the prostate component of the Prostate, Lung, Colorectal and Ovarian Cancer Screening Trial (PLCO, USA), it was concluded that the rate of PCa-related deaths was not significantly different between the screening and non-screening group after 13 years follow up [34]. Hence, appropriate implementation of PSA screening is a highly debated subject. In 2008 the US Preventative Services Task Force (USPSTF) recommended against annual PSA screening for all men > 75 years and in 2012 recommended against screening in any men, with the conclusion that the benefits of screening do not outweigh the harms [35]. Subsequent to these recommendations, Jemal et al*.* examined trends in PCa and found that both incidence and screening rates have declined in the US [35]. The European Urology Association (EUA) still advocate PSA screening but not unless men have been counseled on the potential risks and benefits, and it is only recommended for men who are at an elevated risk of getting PCa [36].

Researchers believe that the limitations of PCa with regards to its specificity, can be overcome by the incorporation of additional clinical and molecular measurements [37, 38]. Results from the Stockholm 3 study (STHLM3) support this idea. This study was initiated to assess the value of incorporating additional blood-based measurements and clinical variables as part of an improved PSA screening model. Here it was shown that a combination of plasma protein biomarkers (PSA, fPSA, hexokinase 2 (hK2), microeminoprotein beta (MSMB), and macrophage inhibitory cytokine 1 (MIC1)), genetic polymorphisms and clinical variables (age, family history, previous prostate biopsy and prostate exam) performed significantly better than PSA alone for detection of PCa. Indeed it was proposed that this model—the STHLM3 model—could lead to reduced PCa mortality with substantially fewer biopsies and reduced over diagnosis (see Table 1) [39]. This view that established diagnostic techniques can be enhanced with incorporation of additional clinical measurements is central to ongoing efforts to develop new biomarker ‘signatures’ as clinical tests, as detailed in the upcoming sections.

**Table 1:** STHLM3 model for prostate cancer screening

STHLM3 model [39]	
Blood proteins	PSA, fPSA, intact PSA, hK2, MSMB, MC1
Genetic polymorphisms	232 SNPs
Clinical variables	Age, family history, previous prostate biopsy, prostate exam

### Digital rectal examination of the prostate

Digital rectal examination (DRE) remains the primary test for initial clinical assessment of the prostate and is recommended based on elevated PSA levels (Fig. 2) [40]. During a DRE, the doctor inserts a lubricated finger through the rectum to feel the exposed surface of the prostate gland. An ‘abnormal’ DRE result is reported if signs of prostate enlargement or growths are felt. Because PCa first materializes as a nodular swelling on the surface of the prostate gland, an abnormal DRE is considered a strong indication of the presence of PCa [10]. Prior to the discovery of PSA, DRE was used as a screening test for PCa although it is now regarded a highly imperfect clinical tool [41]. A significant disadvantage to DRE is that it is subject to inter-examiner variability [42]. It reportedly ‘misses’ a large number of cancers and can only successfully diagnose cancer at a more clinically advanced stage [40, 43]. Nevertheless, DRE is still found to add significantly to information on PCa risk when evaluated in conjunction with other clinical parameters such as PSA [44]. In fact, even though the majority of malignancies identified by DRE are ultimately upstaged, DRE is associated with an increase in the detection of clinically localized tumours. Because it is an inexpensive examination and easy to perform in the clinic, it will remain included in PCA screening protocols [41, 45].

### Prostate biopsy

Definitive diagnosis of PCa is based on a prostate biopsy. Since the landmark paper by Hodge et al*.* in 1989 [46], transrectal ultrasound (TRUS) guided biopsy has become the accepted standard in PCa diagnosis worldwide. TRUS allows imaging of the prostate and seminal vesicles and is used to guide core needle biopsies either through the rectum (transrectal biopsy), through the urethra (transurethral biopsy) or through the area between the anus and scrotum (transperineal) [40]. TRUS-guided biopsies are recommended for men who have a suspicious DRE and/or elevated or rising PSA levels [47]. Transrectal biopsies are most commonly performed. Transperineal biopsies offer greater access to peripheral zones of the prostate, however, they are more invasive and are associated with greater risk of infection [47]. The standard biopsy approach uses an 18 guage needle to obtain between 10 and 12 1.5 cm tissue cores symmetrically throughout the prostate. These are then viewed under a microscope by a pathologist to assess the presence and grade of disease [40]. In many men, initial biopsies appear negative. Multifocal cancers with little clinical significance are also frequently detected. Hence, the chances of misdiagnosis based on tissue biopsy can be as high as 35%. Repeat biopsies are carried out in the event of rising and/or persistently high PSA levels and a suspicious DRE [48]. Generally patients will be diagnosed with a higher grade of PCa upon their second biopsy.

**Fig. 3:**
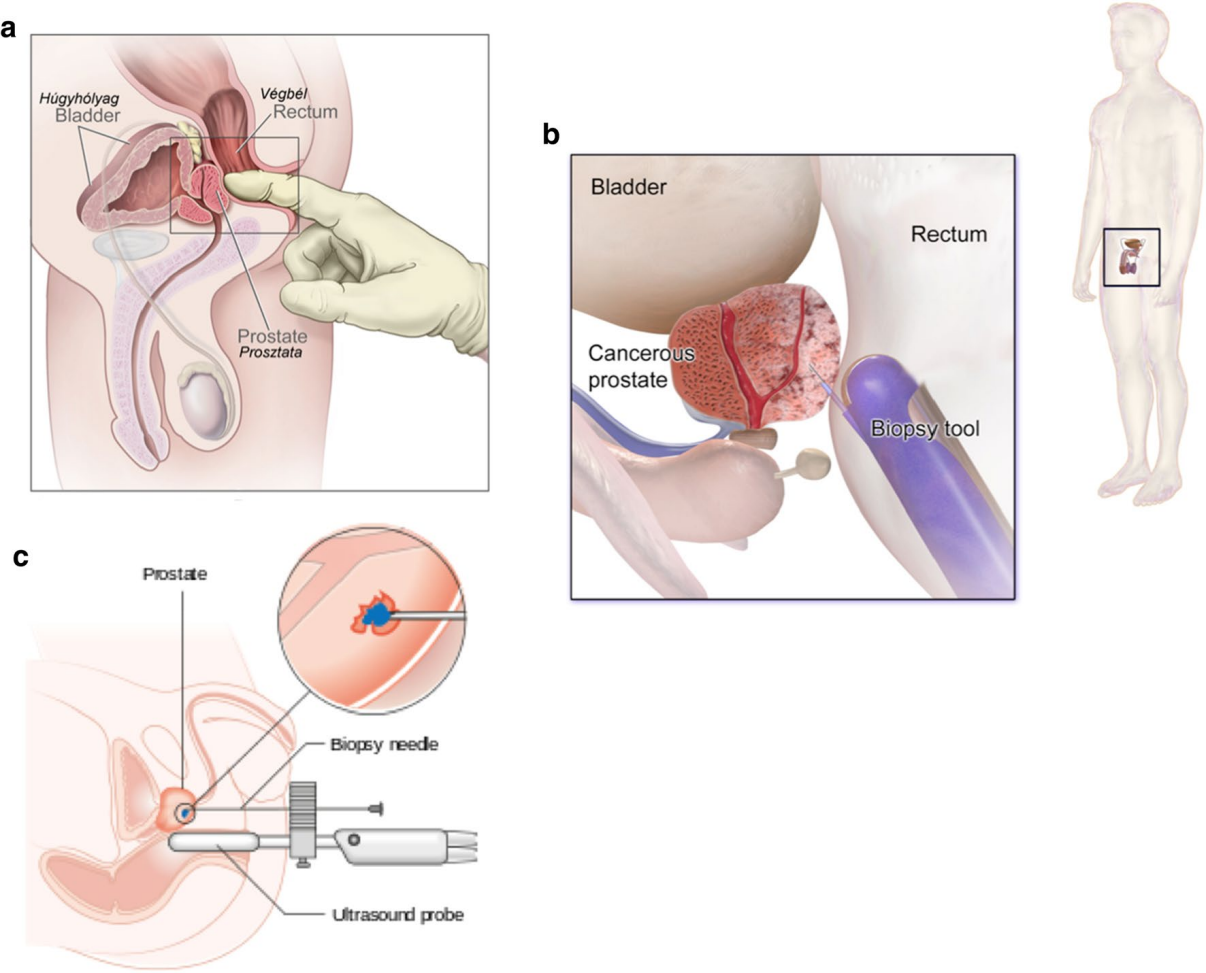
**Diagnosis of Prostate Cancer**. Digital rectal exam is a key component of the physical assessment of the prostate gland (**a**). Men with a DRE that is indicative of PCa are recommended for transrectal ultrasound-guided biopsy (TRUS) (**b**). Up to 12 cores of tumour tissue are sampled using real-time ultrasound imaging to guide the needle to suspected tumour regions. The false negative rate can be as high as 35% in first time biopsies. MRI-TRUS fusion fuses pre-biopsy MRI images with real-time ultrasound imaging using a digital overlay to accurately guide the needle towards previously delineated regions of suspected tumour (**c**)

To improve diagnostic accuracy, magnetic resonance imaging (MRI) is now routinely used to visualize the prostate. Its use in detecting pathology of the prostate gland was first reported by Hricak et al.in 1983 [49]. MRI has since been shown to have a high degree of accuracy in detecting clinically significant PCa [50–53]. Indeed the European Society of Urogenital Radiology (ESUR) argue cogently that MRI should be an integral part of PCa diagnosis and treatment [54]. A number of methods have been explored for the application of MRI to guide prostate biopsy [55]. The MRI-based technique showing the most promise for tissue-based diagnosis is MRI-TRUS fusion (Fig. 3). This involves sampling lesions suspicious for PCa that have been identified in a pre-biopsy MRI. Images from this MRI are stored in the ultrasound device and fused with real-time ultrasound images using a digital overlay. This image fusion provides a 3D reconstruction of the prostate that allows the needle to be accurately aimed towards target regions previously delineated by the radiologist [51, 55]. MRI-methods of sampling have been associated with a number of benefits; fewer men are sampled overall, a greater number of men who have significant PCa are biopsied and fewer of those biopsied receive a diagnosis of insignificant PCa [50]. Irrespective of the biopsy method used, grading of PCa biopsy tissue samples follows the Gleason grading system.

### Gleason scoring

**Fig. 4:**
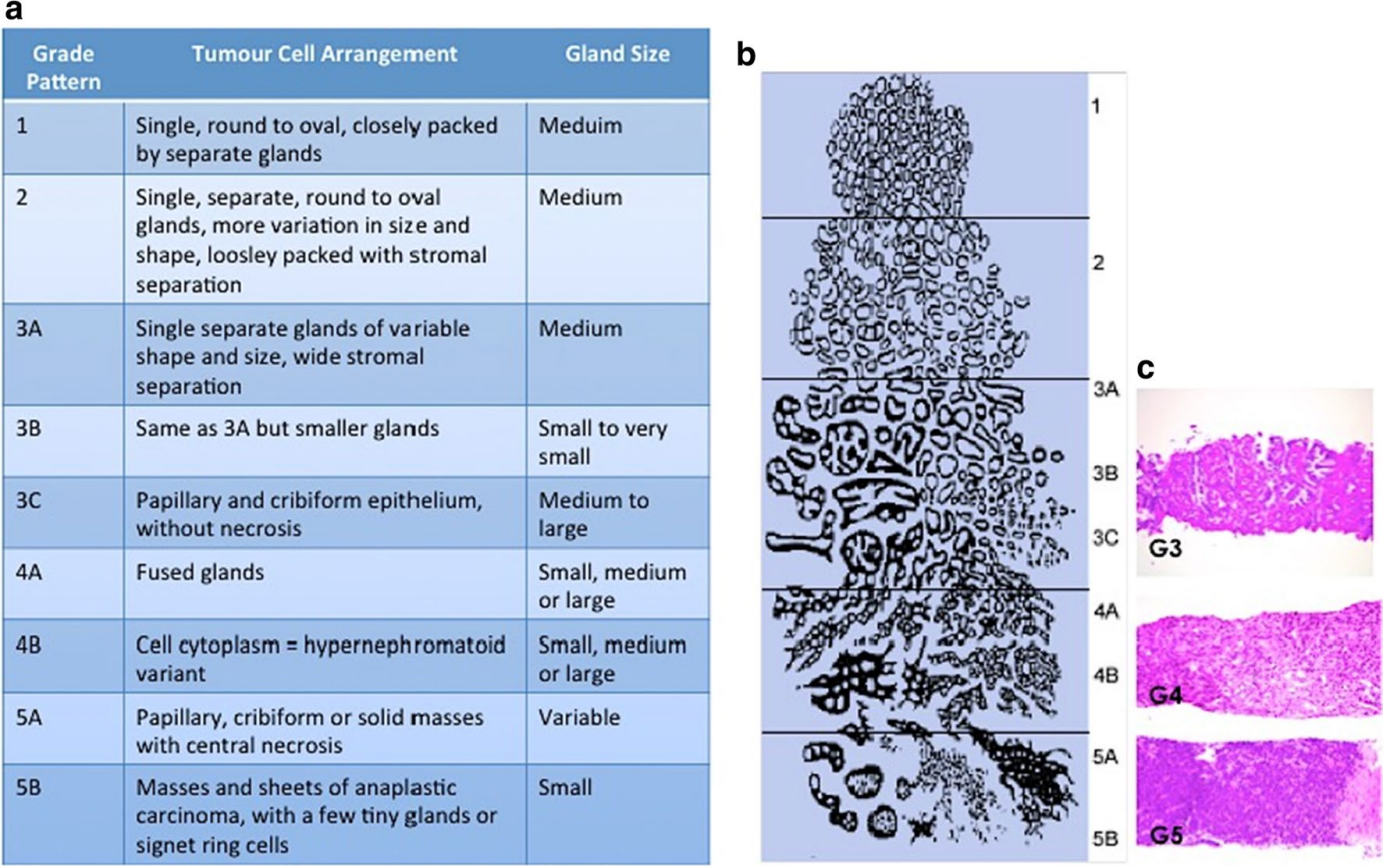
**The Gleason Scoring System**. Pathologists evaluate prostate biopsy samples and ‘grade’ tumour regions based on defined architectural patterns (**a**). Regions of Gleason grade 3, Gleason grade 4 and Gleason grade 5 tumour as observed under the microscope are shown (**b**). Image is adapted from Humphrey et al. [56]

The Gleason grading system uses five basic grades (1–5) that describe different tumor growth patterns and are used to generate a histologic score ranging from 2 to 10 (Fig. 4). This score is achieved by addition of the two most common grade patterns in prostatectomy samples and the most common grade and highest grade in biopsy samples. This is unique in cancer grading as most other malignancies use the single worst grade pattern observed to determine a patient’s disease outcome [56]. This grading system has been used since the 1970s, however, it is now accepted that the original assignment of cancer stage based on Gleason Score (GS) is not appropriate for accurate staging of PCa tumors. For one thing, grades 1 and 2 are never diagnosed in modern pathology practice since the advent of immunohistochemistry. Gleason scores of ≤ 5 therefore represent a redundant group. Secondly, the system does not recognize the multifocal nature of PCa and it has been observed that individual tumour foci within the same prostate specimen can have at least one Gleason grade pattern that differs from the overall Gleason grade of that specimen [57]. Another significant limitation to the GS system is that it is subjective; there is considerable inter-examiner variability in assigning the overall GS for a tumour [58]. There is often significant discordance between the GS given to biopsies acquired pre and post radical prostatectomy (RP) with many patients receiving a higher GS following RP [59]. This is largely due to the ambiguity surrounding a GS of 7—many studies have shown that patient outcomes will vary based on whether their GS of 7 represents a tumor that is mostly GS 4 tumour with some GS 3, or vice versa [60–62]. A modified version of the GS system has therefore been introduced in which PCa tumors are graded as follows: grade group 1 (GS ≤ 6), grade group 2 (GS 3 + 4), grade group 3 (GS 4 + 3), grade group 4 (GS 8) and grade group 5 (GS 9–10). This revision of the GS system has reportedly resulted in more accurate grading of PCa tumours and provides greater reassurance for patients diagnosed with GS 6 PCa that their disease is considered ‘low risk’ [63, 64]. The GS system, even in its modified form, does not fully account for other unique pathological features of PCa, which are thought to influence clinical outcome. Intraductal carcinoma of prostate (IDC-P) is a rare pathological pattern in PCa that, although not considered in the GS system, has been associated with resistance to treatment for ‘’non-organ confined” PCa [65]. Although cribiform carcinoma (CR) and IDC are two separate pathologic entities they are likely to be related on both a pathological and biological level. Indeed studies have shown that men with CR/IDC-negative GS7 PCa have similar survival probabilities to patients with GS6 PCa [66]. PCa is further divided into stages based on ‘TNM Classification of Prostate Cancer’. TNM staging is designed to classify PCa by anatomical extent, as determined clinically and histopathologically [67]. The TNM classification system for describing the anatomical extent of PCa is based on three main components: T = the extent of primary tumour, N = the absence/presence and extent of regional lymph node metastasis and M = the absence/presence of distant metastasis. The addition of numbers to these components further describes the extent of the malignancy [67, 68].

### Treatment options for prostate cancer

Levels of PSA, Gleason score and TNM stage are combined to classify patients according to their level of disease risk and thereby assist in determining the most appropriate treatment strategy [24, 69]. Low risk PCa is considered non-life threatening and so patients can be recommended for ‘watchful waiting’ or ‘active surveillance’. Historically, ‘watchful waiting’ (observation) was recommended for older men with a naturally short life expectancy who would not be suitable for radical treatment. This approach follows patients until their cancer progresses to a point whereby the patient requires palliative treatment [70, 71]. Active surveillance (AS) involves closer monitoring of cancer progression, looking for indications for curative intervention based on regular PSA tests, DRE and repeat biopsies [72, 73]. Curative treatment options for intermediate and high risk PCa include hormone therapy, radical prostatectomy and radiotherapy [74]. Of these, radical prostatectomy and radiotherapy are the two main first-line treatment options for organ confined PCa—usually followed by androgen (hormone) deprivation therapy (ADT) [37, 74]. As PCa is increasingly being diagnosed at an early stage, the non-invasive option of active surveillance is now advocated in place of surgery and radio/hormone therapy, as these are associated with significant side effects.

### Radical prostatectomy

Approximately one third of patients diagnosed with PCa undergo radical prostatectomy (RP) i.e. removal of the prostate gland, in the early stages of their disease [75, 76]. It is broadly considered to be an effective and non-life threatening treatment option for patients with localized PCa. This is supported by the low mortality rate of less than 0.3% for men with intermediate to high-risk PCa who are treated by RP [77]. In reality, however, the procedure is associated with significant side effects that often impact negatively on a man’s quality of life. These common side effects include; impotence, orgasmic dysfunction, incontinence, pulmonary emobolism, rectal injury, urethral strictures and the need for transfusion [78]. More than 50% of men are at risk for ejaculatory dysfunction, which has been cited as the primary concern of men receiving treatment for PCa [79]. Treatment of PCa is further complicated by compounding factors and co-morbidities that are associated with increased age (e.g. cardiovascular disease and diabetes mellitus). In the past, RP, although a relatively straightforward procedure, would not have been considered for men aged ≥ 70 years who would have been presumed to have a life expectancy of less than 10 years. Although this is no longer the case, outcomes for older men who undergo RP are not as promising as for younger men (< 60 years old) [80, 81]. Another form of surgical intervention is cryotherapy. This technique involves destruction of tumour tissue by subjecting it to non-vitally low temperatures. The indications for cryotherapy (cryosurgery) in PCa are vague, however, both the AUA and the EUA agree that it should be an option for patients who do not desire or would not make good candidates for conventional RP. In a ‘salvage setting’ (incidences of increasing PSA following primary curative treatment), salvage cryosurgery is thought to do better than salvage RP as it is associated with reduced morbidity and is less technically challenging [82].

### Radiation therapy

Radiotherapy (RT) is used as a main treatment modality in men with PCa. It can be included as an alternative to surgery although it is more often administered post-operatively, either alone or in combination with hormone therapy (CHRT), depending on the stage of disease or the patient’s preference [83]. The molecular basis of RT is to destroy cancer cells by damaging their DNA. Clinically, the major advantage of RT is its ability to directly attack tumours that are inaccessible for surgical removal. RT is also more selective than chemotherapy as the ionizing beams are focused directly at the tumour and so the entire body does not need to be exposed to a cytotoxic agent [83].

External beam radiation therapy (EBRT) is the most established RT-based treatment option for PCa. EBRT is suitable for all PCa patients of all risk levels and involves daily treatments with a ~ 70 Gy dose of radiation over a period of 7–8 weeks [84]. The success of EBRT for treatment of both clinically localized and advanced PCa is enhanced when combined with hormone therapy. There has recently been interest in stereotactic body radiation therapy (SBRT) for PCa. This method aims to deliver the equivalent of 78 Gy administered during EBRT to the prostate tumour in 2 Gy fractions delivered over 5 days, while sparing the rectum and bladder [85]. This is thought to be a more appealing option for men who are reluctant to undergo the standard 7–8 week course of EBRT and are not suitable candidates for brachytherapy [85, 86].

Brachytherapy involves the ultrasound-guided implantation of radioactive seeds inside or adjacent to the cancerous tumour [87]. For early stage low-intermediate risk PCa, low dose rate (LDR) brachytherapy is considered an effective treatment option, either alone or in combination with EBRT. In this instance, the radioactive seeds are permanently implanted [84]. High-dose rate (HDR) brachytherapy is considered a safe and effective treatment for intermediate-high risk PCa. HDR brachytherapy treatment is administered routinely in 10 min sessions through a temporary catheter that contains the radioactive seeds [84, 88]. Both LDR and HDR brachytherapy are considered the most cost effective PCa treatment regimes and are associated with only minor toxicity [84].

Image guided RT (IGRT) was introduced with the objective of increasing the precision and accuracy of radiation delivery directly to the tumour. Imaging methods include planar imaging, cine-imaging, volumetric imaging, marker localization, marker tracking, surface matching and surface tracking [89]. Treatment delivery methods for IGRT include three dimensional conformal RT (3D-CRT) and intensity-modulated radiotherapy (IMRT) [90]. IMRT uses computer controlled linear acceleration to deliver precise radiation doses to the malignant tumour or specific areas within the malignant tumour. The computer generates a custom intensity modulation based on the target volume dose and tissue protection objectives pre-defined by the radiation oncologist. This allows the dose to conform more precisely to the 3D shape of the tumour by modulating the radiation beams in multiple small volumes [82, 91].

In the past, the prescribed dose of radiation was generally kept within the range of 64–70 Gy, delivered in fractions of 1.8–2 Gy, however, clinical trial data has since indicated that this dose is insufficient for disease control [92]. The advent of more precise RT techniques means that dose escalation regimes are now achievable, effective and safe. Dose escalation can be achieved with 3D-CRT or IMRT or by boosting conventional RT with HDR brachytherapy. Multiple retrospective studies have indicated that increases in the total dose of radiation by up to 10 Gy is associated with improved rates of recurrence free survival (from 50%–70%) with minimal increase in toxicity [84, 86, 92].

In cases of disease recurrence, salvage radiotherapy (SRT) is considered the only potentially curative therapy available. Indeed the American Society for Therapeutic Radiology and Oncology—American Urological Association (ASTRO/AUA) recommend SRT to all men with biochemical recurrence, even without clinical evidence of distant metastasis [93]. Toxicities of RT affect both the gastrointestinal and genitourinary regions and may manifest with incidences of nausea and diarrhea. Unfortunately, it is difficult to predict the degree to which an individual will suffer from such effects [94].

### Hormone therapy

Androgen deprivation therapy (ADT) is the recommended first line treatment in all men with high risk or metastatic PCa. Nearly 50% of men with PCa will receive ADT at some time following diagnosis and the majority of these will undergo ADT for at least 2 to 3 years [95, 96]. The goal of ADT is to deprive the PCa tumour of androgens, which are the hormones that drive prostate epithelial cell growth and proliferation. There are numerous classes of ADT drugs available. The most commonly used are those which target either androgen production or androgen receptor activation [97]. Common adverse effects associated with ADT include fatigue, hot flushes and impotence. ADT also increases the risk of heart disease and the prevalence of metabolic syndromes in men with PCa [96]. These side effects are significant considering the length of time that a patient would spend receiving regular ADT. Several randomized trials have reported significantly better long-term survival in patients treated with combined hormone and radiation therapy (CHRT) [98, 99]. However, the precise duration of hormone ablation therapy required to be effective remains unclear and can range from 3 months to 3 years [100].

### Chemotherapy

For men with recurrent or advanced PCa, treatment options that target the prostate gland alone are insufficient. Chemotherapy treatment with taxanes has been shown to improve survival in patients with metastatic PCa. In particular, docetaxel and cabazitaxel have become the standard first and second-line chemotherapeutic agents of choice for patients that have failed hormone therapy [101]. Mechanistic studies have indicated that the advantage of using taxanes as opposed to other chemotherapeutic agents might be through their indirect effects on the androgen receptor [102]. It has thus been suggested that earlier use of chemotherapy in patients who are sensitive to hormonal therapy could improve efficacy and tolerability with a greater impact on clinical outcome [103]. A number of large-scale Phase III clinical trials, evaluating the combined use of chemotherapy with ADT versus ADT alone, have been reported. The GETUG-AFU trial, which was set up across 29 centres in France and Belgium, was the first to report observations from combined ADT and docetaxel treatment. With a median follow up of 50 months per patient, this study found that there was no significant increase in survival for patients treated with docetaxel + ADT as opposed to those treated with ADT alone. Moreover, this study reported a number of serious adverse effects, including four treatment related deaths in those patients treated with docetaxel + ADT. As such, this study concluded that docetaxel should not be recommended as a fist line treatment for patients with non-castrate metastatic PCa [104]. Similarly, the CHAARTED trial was designed to compare overall survival rates for men with metastatic, hormone sensitive PCa who received 6 cycles of docetaxel at the beginning of ADT, versus men who received treatment with only ADT. In contrast to the GETUG-AFU trial, this study reported significantly longer overall survival for men treated with ADT + docetaxel, with a more pronounced clinical benefit observed in patients who had a greater disease burden. The authors therefore conclude that docetaxel should be recommended as a first-line treatment option for men with metastatic hormone sensitive PCa [105]. STAMPEDE represents the largest (~ 3000 men enrolled) trial to date that has been set up to evaluate the benefits of combined chemotherapy and hormone therapy. As well as docetaxel, this study also investigated the combination of zoledonic acid with standard ADT. Based on the findings reported, it was concluded that zoledonic acid showed no evidence of improved survival and was also accompanied by an increase in adverse effects. However, docetaxel did offer a substantial improvement on overall survival. This study concluded that docetaxel should be recommended for adequately fit men who are set to commence ADT [106]. The most significant caveat to chemotherapeutic intervention at an early stage is the risk of toxicity, which in some cases can cause death—as observed in the GETUG-AFU trial [104]. The challenge therefore, is to be able to select only patients who are most likely to benefit from chemotherapeutic treatments [101]. Currently there are no adequate biomarkers to guide appropriate patient selection for early chemotherapeutic intervention. The protein SLCO1B3 has been suggested to have a role in the development of anticancer chemotherapy resistance in multiple cancer types including PCa [107]. Further elucidation of the functional role of SLCO1B3 may lead to novel therapeutic strategies for treatment of advanced chemo-resistant PCa [107].

### Immune therapies

Immune therapies are not so widely used for treatment of PCa as for other cancer types, although it continues to be an area of active research [108]. Sipileucel-T (Provenge ®) is an FDA approved autologous vaccine that is derived from ex vivo culturing of patients peripheral blood mononuclear cells with antigen presenting cells. It is designed to target prostatic acid phosphatase (PAP) and granulocyte–macrophage colony stimulating factor (GM-CSF), which are found in the prostate epithelium and promote cancer growth [109, 110]. Clinical trials have shown that the vaccine contributes moderately to overall survival and offers a slight reduction in mortality risk, although the greatest disease benefit is observed in patients with low disease burden [109]. The treatment is generally recommended for minimally-symptomatic CRPC patients, however, the vaccine is considered to be prohibitively expensive [108, 109]. Another vaccine that has been trialed in PCa patients in PSA-TRICOM (Prostvac®). The vaccine consists of two vectors—a priming agent and a boosting agent—both of which contain PSA and immune-stimulating molecules (B7-1, ICAM-1 and LFA3). The vaccine is well tolerated and seems to confer a modest survival benefit in asymptomatic CRPC patients. There is some evidence to suggest that the survival benefit from use of this vaccine will be accentuated if used in combination with chemotherapy, and this is currently being explored in clinical trials [109, 111]. Chimeric antigen receptor (CAR) T cells are a type of cellular therapy, whereby patients are injected with autologous T-cells that have been engineered *ex-vivo* to express a chimeric antigen receptor directed against a tumour-associated antigen [109]. For PCa treatment, the cells target prostate specific membrane antigen (PSMA). This therapy is only in the early stages of clinical evaluation (NCT01140373). Checkpoint inhibitors are a commonly used immune therapy that have shown significant therapeutic benefit in the treatment of metastatic melanoma (ipilimumab) and more recently in treatment of advanced non-small-cell lunch cancer (nivolumab). These drugs target the PD-1 signalling pathway in order to promote T-cell activation and modulate the immune response against cancerous cells [109]. In a previous study, treatment of PCa patients with iplilimumab did not result in an overall survival benefit [112]. Currently, a checkpoint inhibitor called Pembrolizumab is being investigated in a phase II study for treatment of metastatic CRPC after treatment with ADT (NCT02312557).

### Current prostate cancer biomarkers

As described in previous sections, the lack of specificity of PSA as a marker for PCa makes it a poor biomarker for prediction of disease recurrence, which affects a significant number of men with PCa [113]. Efforts to improve upon PSA were initially addressed by attempting to identify and measure additional isoforms of PSA. Free PSA (fPSA) is the small amount of PSA that is not bound to serum proteins and the percentage of fPSA has been used to stratify men with total PSA levels of 4–10 ng/ml and a negative DRE into PCa risk categories. A meta-analysis has shown that measurement of the percentage of fPSA improved diagnostic performance among men with total PSA in the range of 2–10 ng/ml, compared with total PSA alone [114]. However, fPSA can produce conflicting results as levels are also elevated in men with BPH and prostatitis [115]. Both PSA velocity (PSAV) and PSA doubling time (PSADT) have been used to measure the change in PSA per year and specific value increases in PSA, respectively. These measurements are also considered to increase the specificity of PSA [115]. Measurement of an isoform of proenzyme PSA called [-2] proenzyme PSA (p2PSA) has also been reported to enhance the specificity of PSA-based screening [116]. The Prostate Health Index (phi) is an immunoassay-based test that combines measurements of several forms of PSA in blood—PSA, fPSA and p2PSA—as part of an algorithm, which provides a personalized PCa risk assessment for the patient [117]. The phi test has been shown to provide better specificity for PCa diagnosis than any of the forms of PSA alone, and is one of few new tests to have achieved FDA approval in the US [117]. Although measurements of PSA isoforms appear to be of use, they are still more ‘prostate specific’ than ‘cancer specific’. Hence, the phi tests cannot be used to stratify patients based on PCa risk, which makes the identification of clinically significant disease difficult [118].

### Tissue-based prostate cancer biomarkers

The prognostic value of the protein Ki-67 has been well documented. This tissue-based marker has been shown to be a significant determinant of distant metastasis and PCa-related death [119–121]. In addition, phosphatase and tensin homologue (PTEN) loss has also been found to add prognostic value to Gleason score, PSA and Ki-67 tissue staining [122]. PTEN loss is routinely observed in prostate tumors with high Gleason grade, although it is recommended that it would only be of real use as a biomarker if combined with a panel of additional markers. Currently there are no PTEN or Ki-67 assays available that meet the standards required by the European Commissions for in-vitro diagnostics (CE-IVD) [123].

A number of tests have recently emerged, which claim to better predict PCa occurrence based on the observed expression of multiple genes/proteins. One example is the Decipher test offered by Genome Dx Biosciences. This is a gene-based classifier containing 22 non-coding RNA sequences that was both developed and verified in fresh frozen paraffin embedded (FFPE) tumor tissue specimens. This test uses a whole-transcriptome microarray assay for analysis of gene activity in PCa FFPE specimens [124]. The expression of these gene markers is used to calculate the probability of clinical metastasis within 5 years of radical prostatectomy, and within 3 years of biochemical recurrence [125]. The test can also offer risk assessment to help tailor treatment options for patients diagnosed with localized prostate cancer on biopsy.

A similar test—the OncotypeDX offered by Genomic Health Inc.—measures a 17-gene signature as an independent predictor of adverse pathology in PCa. The signature is comprised of 5 reference genes (for normalization) and 12 cancer genes, which represent biological pathways with a known role in PCa progression; the androgen pathway, cellular organization pathway, proliferation pathway and stromal response pathway [126]. This test was developed in a bid to address the impact of tumor sampling in predicting aggressive PCa i.e. by overcoming the inherent genetic variations between regions of individual tumors and the limited tumor material acquired by needle biopsy [127]. Oncotype Dx is most applicable for men with newly diagnosed, early stage PCa and is used to determine the need for treatment [128]. The RT-PCR-based assay has been clinically evaluated for prediction of high grade and/or non-organ confined PCa at radical prostatectomy using biopsy samples containing as little as 1 mm of tumor tissue [125, 126].

Recently, a test based on the expression of cell cycle progression genes in primary tumor samples has shown great promise in accurately stratifying patients with localized PCa according to disease aggressiveness. The ‘Prolaris’ test (Myriad Genetics Inc.) is a genomic test for predicting PCa aggressiveness in conjunction with clinical parameters such as Gleason Score and PSA [125]. This RNA expression-based assay directly measures tumor cell growth characteristics. The test combines the gene expression levels of 31 cell cycle progression (CCP) genes and 15 house-keeping genes to give a CPP score [129]. This assay has since been evaluated in numerous cohorts representing disparate patient populations using both ‘fresh’ tumor biopsy samples and sample sections that have been prepared for long-term storage in paraffin wax [130–133]. It is envisaged that this test will be most applicable in helping to identify low-risk patients who can be safely managed with active surveillance [24]. Recent reports indicate that the Prolaris test can also be used to predict biochemical recurrence in post-prostatectomy patients [128].

The ‘ProMark’ assay (Metamark), is a protein based prognostic test for predicting PCa aggressiveness—particularly for patients with Gleason grade 7 disease [125]. This assay measures 8 protein markers using a multiplexed in situ imaging system [134]. The test has been shown to reproducibly provide simultaneous quantification of protein levels and functional activities using tissue specimens [135]. The intended use of this test is to supplement current biopsy-based PCa risk assessment methods in cases where a clinical decision regarding active surveillance versus active treatment is not straightforward. PCa is a highly heterogeneous and multifocal disease and so, the 8 biomarkers which comprise the ProMark assay have been specifically selected and evaluated to predict pathology outcome regardless of whether they are measured in low or high grade tumor specimens from the same patient [136].

Although the molecular signatures described here are indeed promising, tissue heterogeneity is a significant complicating factor for reliable biomarker measurement. Because PCa is a multifocal pathology and only a small proportion of the prostate is sampled during biopsy, the most aggressive areas of tumour are frequently either over or under-sampled [137–139]. For a disease that generally remains present for such a long time, samples that are more amenale to routine, minimally-invasive testing are more desireable.

### Fluid-based prostate cancer biomarkers

Gene-based assays have, to date, made much more progress than protein-based assays in efforts to identify suitable fluid-based biomarkers. The expression of a gene called DD3PCA3, which codes for a protein called Prostate Cancer Antigen 3 (PCA3), has been shown to correlate with malignant PCa. Indeed, it has been demonstrated that PCA3 mRNA is not at all expressed in normal prostate tissue and expressed at very low levels in BPH specimens [137]. Moreover, the expression of PCA3 can be measured in urine. The Progensa assay compares the concentration of PCA3 mRNA levels to PSA mRNA levels to produce a urinary PCA3 score [140]. It has been found that urinary PCA3 scores (PCA3-mRNA/PSA-mRNA) are consistently superior to serum PSA levels for diagnosis of PCa. Unlike PSA, PCA3 expression remains constant during BPH and prostatitis, thereby making it more sensitive than PSA for detection of PCa. It has therefore been suggested that the PCA3 score be used as an exclusion tool [141, 142]. Although PCA3 mRNA measurements can be made using urine passed without the need for prostatic massage, a downside to this test is that it can only be performed using the first 20–30 mL of urine voided after a DRE. As such, valid results are only achieved in approximately 80% of cases [141]. The measurement of PCA3 has also been combined with another well-known biomarker of PCa—the TMPRSS2:ERG gene fusion—as part of the Mi-Prostate Score [143]. TMPRSS2 is an androgen-regulated gene that is overexpressed in PCa tissue and plays a key role in cancer cell invasion and metastasis. Fusion of TMPRSS2 with ERG occurs via chromosomal rearrangement and is associated with poor prognosis in PCa [144]. Both the PCA3 and TMPRSS2:ERG biomarkers can be detected in patient’s urine after DRE, which provides the basis for a non-invasive, easy to use clinical test. The Mi-Prostate Score incorporates blood PSA levels with urinary levels of PCA3 and TMPRSS2:ERG to allow for stratification of PCa while avoiding unnecessary biopsies [125, 143, 145].

A newly available urine test from the same team who developed the PCA3 assay is SelectMDx (MDx Health). It measures expression of HOXC6 and DLX1 genes in urine using KLK3 (PSA) as an internal reference. This test was designed following quantitative PCR analysis of both tissue and urine samples, which led to the identification of 8 urinary biomarkers for PCa. This was subsequently refined into a 3-gene panel—HOXC6, TDRD1 and DLX1—that is measurable in urine [146]. This urinary 3-gene panel has shown higher accuracy in detecting aggressive (Gleason > 7) PCa compared to the Progensa PCA3 assay [147]. Subsequently, two prospective multicenter studies were conducted to validate the gene panel in whole urine and develop a model combining molecular profiling with traditional clinical risk factors. The risk score derived from combining the two most promising gene markers (HOXC6 and DLX1) with PSAD, DRE and PSA was found give the most accurate detection of high grade PCa upon biopsy and was also successfully validated in another independent patient cohort [146]. As yet, this is not an FDA approved test, although it has been CLIA-accredited. Cost effectiveness studies have revelaed that incorporation of the SelectDx test into clinical assessment of PCa resulted in a saving of €128 ($143) and a gain of 0.25 in patient quality of life years, compared to using only PSA to select patients for prostate biopsy [148].

PCA3 has also been incorporated into a new test called the ExoDx Prostate Intelliscore, which is offered by ExosomeDx. This test involves analyses of exosomal RNA for three biomarkers—PCA3, TMPRSS-ERG and SAM pointed domain containing ETS transcription factor (SPDEF)—which are known to be expressed in men with high grade PCa [149]. It has been shown that addition of this test to standard clinical variables (PSA, age, race and family history of PCa) improves discrimination between low-grade (Gleason 6) and high-grade (Gleason ≥ 7) PCa [150]. The ExosomeDx Prostate test aims to reduce the number of unnecessary biopsies and is now available in the US as a CLIA-approved clinical laboratory-developed test (LDT).

Another urine test, Prostarix (Metabolon Inc.), uses metabolomics technology to measure levels of 4 amino acids associated with PCa. Using liquid chromatography and mass spectrometry coupled with a logistic regression algorithm to generate a score, the test claims to aid the assessment of cancer detection and can be used to distinguish between benign prostate, clinically localized PCa and metastatic disease [150].

Although urine is an easily accessible sample for biomarker measurements, some of the urine-based assays require urine that is voided immediately following DRE, which is an invasive procedure. Moreover, the collection of urine cannot be fully controlled and so sampling variability must be considered. Blood, on the other hand, is also easily accessible and collected under much more controlled conditions. Successsful clinical research on serum-based biomarkers for PCa detection remains confined to the kallikrein field. A four prostate-specific kallikrein panel has shown great promise as a serum-based test for PCa. The 4Kscore is a combined measurement of total PSA, fPSA, intact PSA and human kallikrein-related peptide 2 (hK2). It has been observed in multiple studies that the serum 4Kscore assay accurately predicts the risk of biopsy-detectable high-grade PCa in men who have not undergone a prostate biopsy [151]. Indeed, one study showed it to be more predictive of PCa than PCA3, and it was therefore recommended for use alongside PCA3 for detection of PCa in pre-screened men [152]. The 4Kscore is now commercially available in the US as a CLIA-approved laboratory developed test (LDT) and although not (currently) FDA approved, it appears to have some clinical utility [140]. The current tissue-based and fluid-based biomarker tests for PCa along with their recommended use and FDA status are summarized in Table 2.

**Table 2:** Newly emerging tests for prostate cancer

Assay	Marker description	Assay type	Biomaterial	FDA approved
***Tissue-based***				
Oncotype DX	17 genes	RT-PCR	FFPE needle core biopsy	No
Prolaris	46 genes	RNA expression	FFPE needle core biopsy	Yes
ProMark	8 proteins	Immnofluorescent imaging	FFPE needle core biopsy	No
Decipher	22 coding and non-coding RNAs	Whole-transcriptome microarray	FFPE needle core biopsy	No
Confirm MDx	3 genes	Quantitative methylation-specific PCR	Prostate needle core biopsy	No
PCMT	mtDNA deletions	Quantitative PCR (specific for mtDNA)	Prostate needle core biopsy	No
***Fluid-based***				
phi	PSA, fPSA, p2PSA	Multi-analyte Immunoassay	Serum	Men > 50 with total PSA 4–10 ng/mL and negative DRE
4K score	Total PSA, fPSA, intact PSA, hK2	Multi-analyte Immunoassay	Plasma	No
Progensa (PCA3)	PSA and PCA3 mRNA	In vitro RNA TMA assay	Post-DRE first void urine	Only when repeat biopsy considered
SelectMDx	HOXC6, DLX1, KLK3	Reverse transcription PCR (RT-PCR)	Post-DRE first void urine	No
MiPS	PSA,PCA3 and TMPRSS2:ERG mRNAs	In vitro RNA TMA and Hybrid Protection Assay (HPA)	Post-DRE first void urine	No
Prostarix	4 Amino acids: sarcosine, alanine, glycine and glutamate	Liquid chromatography and mass spectrometry	Post-DRE urine	No
ExoDx prostate (IntelliScore)	Exosomal RNA (ERG, PCA3, SPDEF)	RT-PCR	Urine	No

Table adapted from Falzarano et al. [141]

### Understanding prostate cancer biology for identification of novel biomarkers

To date, none of the tests available have been evaluated in prospective randomized trials and so their optimal indication for clinical use remains uncertain [153]. As such, despite the substantial number of tests available with reported applicability for PCa prognosis, few have been approved by the FDA (Table 2). Moreover, the tests described are only applicable in a diagnostic and repeat biopsy setting. Identifying molecular marker(s) that can function as a non-invasive clinical tool to aid in the management of PCa treatment remains a pertinent clinical need. Identification of such biomarkers does require a thorough understanding of PCa biology in order to guide such investigations.

### The role of androgen signaling

**Fig. 5:**
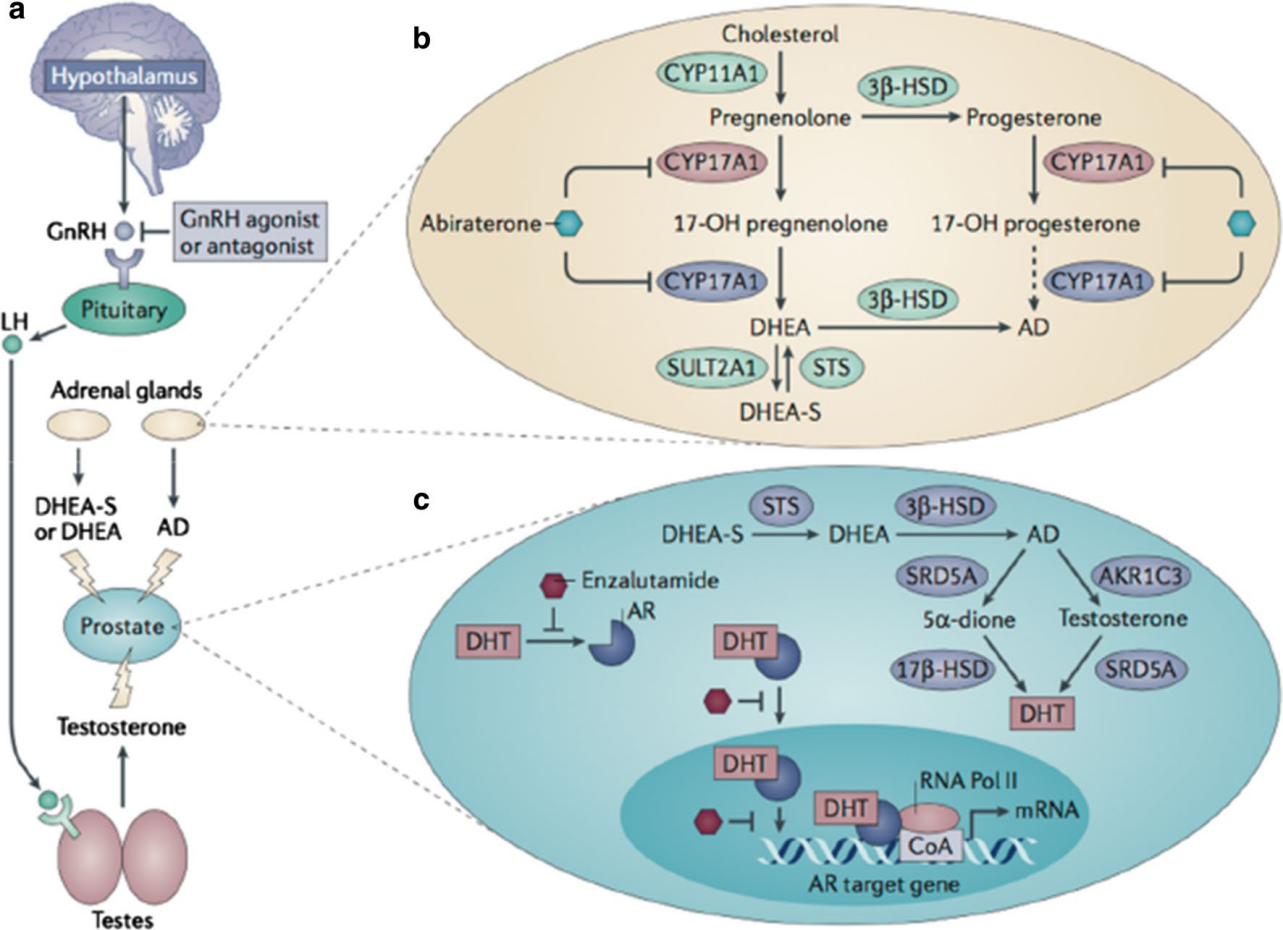
**Androgen Signalling in Prostate Cancer**. Androgen receptor (AR) signalling is regulated by the hypothalamic-pituitary–testicular axis. Androgen production is predominantly regulated by the gonadotropin-releasing hormone (GnRH) and lutenizing hormones (LH), which promote testosterone secretion from the Leydig cells of the testes. GnRH and LH are primary targets for androgen deprivation therapy (**a**). Weak androgens such as androstenedione and dehydroepiandrosterone (DHEA) are also produced by the adrenal glands, which promote de novo steroidogenesis in the presence of elevated levels of cholesterol (**b**). In the prostate androgens are converted to testosterone and dihydrotestosterone (DHT), which bind to and activate AR. Bound AR is translocated to the nucleus where it initiates transcription of AR target genes (**c**) [155, 162] (Figure adapted from Watson et al. [155])

Androgen signaling has an integral role in development and progression of PCa, which is why androgen deprivation therapy (ADT) is a primary treatment option for the disease. However, a significant proportion of PCa patients (~ 25%) progress from being hormone sensitive initially to becoming insensitive to androgen therapy. This is termed castrate resistant prostate cancer (CRPC). Many patients with CRPC will develop cancer recurrence, which generally progresses to metastatic CRPC (mCRPC). Unfortunately, mCRPC is deemed incurable and so greater understanding of the molecular mechanisms that lead to the development of CRPC is crucial for clinical management of the disease [154]. Androgen signalling is regulated by the hypothalamic-pituity-testicular axis (Fig. 5), promoting testosterone secretion from the Leydig cells of the testes [155]. Although, not essential for survival and proliferation of the normal prostate, testosterone (and its derivative dihydrotestosterone, DHT) is essential for prostate tumour growth and progression [156]. As such, the mainstay of conventional ADT are GnRH or LH agonists, which reduce testosterone levels by stable secretion of androgen from the testes [155, 157]. Examples include Enzalutamide and Abiraterone, which have proven effective in improving survival outcomes for patients with metastatic PCa [158]. A critical component of the androgen-signaling axis is the androgen receptor (AR). Rising PSA levels—a main target gene of AR—indicates that AR activity is somehow inappropriately restored, despite either surgical or chemical castration in men with CRPC [157]. It is thus widely accepted that CRPC is neither hormone refectory nor androgen-independent, as previously believed [159, 160].

Mechanisms of castration resistance comprise both re-activation of AR signaling, despite low levels of circulating androgens, and activation of alternative AR-independent pathways [161]. It has been shown that CRPC cells that emerge after ADT have upregulated expression of the enzymes that convert adrenal androgens to testosterone and DHT—the two main physiological targets of AR [157, 162]. Androgens can also be synthesized de novo from cholesterol by cytochrome P450 enzymes [97, 159]. Previous analysis of serum and prostate tissue from PCa patients revealed that DHT levels in prostate tissue and serum post-ADT remained at 25% and 7.5% of the amount measured prior to ADT, respectfully. Such concentrations are sufficient to activate the molecular pathways that drive PCa growth [97, 163].

Another mechanism that has been proposed is amplification of the AR gene. Increased levels of AR are observed in 20–30% cases of CRPC [156] and create a molecular environment that is hypersensitive to androgen stimulation [97]. Mutations in AR are also thought to contribute to aberrant AR signaling and are observed in 10–20% of cases of CRPC. Alternative splicing of AR mRNA is another mechanism implicated in the development of CRPC. AR variants (AR-V) re-establish expression of androgen-regulated genes in the absence of androgen, as well as inducing expression of their own set of targets [97]. Examples of AR-V that have been identified in clinical samples include ARV-7, AR^v567es^ and ARV1. ARV-7 is the best characterized of these, owing to the fact that there are available antibodies, which enable analysis by immunohistochemistry in patient tissue samples [97, 155]. Levels of ARV-7 are typically elevated in tumours that also contain elevated levels of full length AR. It has also been proposed that growth factor signaling through tyrosine kinases plays a role in activating AR by phosphorylation [97, 161].

The fact that there are multiple mechanisms by which AR activity is maintained in the presence of low levels of androgen gives rise to a molecularly diverse group of CRPC tumour cells, even within the one patient [157]. As such AR itself, AR-Vs, AR interaction partners and processes downstream of AR signalling remain viable targets for therapeutic intervention in CRPC [164]. However, increased expression of AR in PCa tumour tissue does not qualify as a biomarker for prognosis and hormonal response, as levels of estrogen receptor do for patients with breast cancer. Nevertheless, chromosomal rearrangements leading to novel fusions between the androgen-regulated promoter of the TMPRSS2 gene to the 3′ end of oncogenic epidermal growth factor (ERG) (TMPSS-ERG fusion) is considered a tissue marker of advanced PCa [157]. AR activity could be better refelected by phosphorylation status of proteins involved in the relevant pathways. Advancements in proteomics technologies has indeed heightened interest in ‘so-called’ phosphoproteomic studies to advance PCa research [165].

### The tumour microenvironment

The survival of cancer cells is believed to be regulated by both inherent cellular responses and the tumour microenvironment itself [166]. Understanding the underlying mechanisms of disease progression will be essential in overcoming treatment resistance and slowing disease advancement in PCa. Conditions within the tumour microenvironment including oxidative stress, hypoxia, nutrient deprivation and low pH, contribute to genetic instability through the induction of increased DNA damage, enhanced mutagenesis and impaired DNA damage pathways [167]. A number of these ‘hallmarks’ of the tumour microenvironment can be attributed to altered cancer cell metabolism, which is referred to as the ‘Warburg effect’. This describes the phenomenon by which cancer cells produce large amounts of lactate through glycolysis—even in aerobic conditions. The glycolytic activity of cancer cells provides an acidic environment that is harmful to normal cells but has no effect on tumour cells. In PCa, increased aerobic glycolysis has been observed in advanced stage tumours, while de novo fatty acid synthesis and increased protein synthesis are common features of both primary and advanced PCa [168].

### Influence of hypoxia and nutrient deprivation in the tumour microenvironment

Tumour cells undergo a variety of biological responses when under hypoxic conditions, including activation of signaling pathways and changes in gene expression patterns that render them able to survive and increase tumour aggression [169]. It has also been reported that when grown under hypoxic conditions, epithelial cells may reprogram toward a more mesenchymal phenotype due to the activation of E-cadherin transcriptional repressors [170]. Hypoxic PCa cells are about three-fold more resistant to the effects of radiation, as DNA-damaging agents require adequate intratumoural oxygen to be maximally effective [171, 172]. This is because the DNA damage induced by ionizing radiation is more readily repairable in the absence of molecular oxygen [173]. Hypoxia can also affect the success of conventional chemotherapy and has been identified as an important factor in the development of chemo resistance [174]. Indirectly, hypoxia can lead to treatment resistance by modifying gene expression and other posttranslational effects. This results in proteomic changes that lead to deviations in cell proliferation and cell cycle dynamics, which ultimately has an effect on the number of cells that can be targeted by radiation therapy or chemotherapy [172]. In PCa, signs of hypoxia and metabolic stress in the prostate tumour tissue are exacerbated following ADT and it has been suggested that androgen withdrawal induces hypoxia in androgen-sensitive tissue [175]. It has also been suggested that the hypoxic microenvironment can enhance the transcriptional activity of the androgen receptor (AR) [169, 176]. Studies have shown that hypoxia is associated with early biochemical recurrence and also local disease recurrence in the prostate gland [177]. Overall, hypoxic conditions are now considered an independent poor prognostic indicator for patients with PCa [178].

In order to survive low oxygen conditions, cancer cells express or overexpress genes that allow them to survive and grow. Recent studies have shown significant hypoxia-induced disruption to the global transcriptome, resulting in the differential expression of many transcriptome factors and their targets [179]. It has therefore been suggested that gene signatures of the transcriptional response to hypoxia could be used to stratify patients in terms of prognosis, predict response to hypoxia-modifying therapies and increase understanding of the complexities of hypoxia in the tumour microenvironment [180]. The most relevant transcription factors that are responsible for the adaption of cells to hypoxic conditions are hypoxia-inducible factors (HIF), especially HIF-1 [181]. This protein stimulates transcription of a series of genes that facilitate the hypoxic response, including vascular endothelial growth factor, erythropoietin and anaerobic glycolysis. However, Hif-1 is unstable and relatively low abundant making it difficult to measure accurately in biological samples. Amongst the genes targeted for up-regulation by the HIF pathway in cancer cells, Carbonic Anhydrase IX (CA IX) generally shows the most dramatic transcriptional activation [182]. CA IX is a more stable protein than HIF-1 and therefore frequently used as a biomarker of hypoxia, however, its expression level does not always correlate with hypoxia as it is also regulated by constitutive Hif-1 α expression and by other transcription factors. Pimonidazole is an exogenous hypoxia marker that has been explored as a biomarker for more aggressve PCa. Due to lack of standardization, this tissue-staining assay is not feasible for routine clinical use, however, the transcriptional activity associated with it’s up-regulation could point towards additional essential genes of biomarker potential [183]. Based on this, Yang et al. have reported a 28-gene signature for hypoxia, which has been shown to be clinically useful in the identification of hypoxic tumours that have poorer outcome. The prognostic utility of this signature was demonstrated in eleven different cohorts of low to high risk PCa patients with localized disease [184]. Recent studies exploring the influence of hypoxia on clinical outcome in PCa have provided a strong rationale for integrating current therapeutic regimes such as RT, with hypoxia-targeted treatment approaches [177]. Hence, gene/protein signatures that are reflective of hypoxic status are likely to be clinically useful in guiding treatment decisions for PCa.

It has been shown that nutrient deprivation, like hypoxia, induces the Warburg effect to support cell viability upon starvation-induced stress. In nutrient deficient conditions, proliferating cancer cells shift their glucose metabolism from oxidative phosphorylation to glycolysis, using intermediates of the glycolytic pathway to synthesize amino acids, lipids and nucleic acids to meet the energetic demands of proliferation [185, 186]. Although energetically unfavourable, this altered metabolism contributes to tumour growth, oncogenic signaling and transformation-associated epigenetic changes [187]. Recent studies have shown that nutrient deprivation, particularly a reduction in supply of glucose, may play a major role in tumour cell tolerance to the oxidative stress encountered within the solid tumour environment. Li et al. have demonstrated that glucose deprivation increases radioresistance of both colon and prostate cancer cells [166, 188]. Under nutrient deficient conditions, cancer cells can scavenge energy precursors and evade cell death through a process called autophagy. Autophagy is a catabolic process that enables cells to obtain energy by recycling amino acids and other intracellular nutrients, thus providing cells with an alternative mechanism to protect themselves against nutrient-deprivation induced stress [189, 190]. The connection between autophagy and cancer cell metabolism is a topic of great interest and potential clinical relevance in cancer research [191]. This is because, when cells are subjected to nutrient deficient conditions, they use an autophagic pathway to simultaneously decrease overall protein synthesis and increase rates of protein degradation [192, 193]. In PCa, it has been shown that alterations in AR activity as result of ADT, can also affect cancer cell metabolism via multiple intra- and extra-cellular signaling pathways. It has been demonstrated that PCa cells can increase their energy supply by taking up energy-rich metabolites from neighbouring stromal fibroblasts, which thereby provides cells with the energy-rich microenvironment required for tumour growth [194]. It is postulated that these metabolic alterations have a role in promoting the progression of PCa to lethal CRPC status [194]. Overall, there are a myriad of metabolism-related enzymes and pathways that warrant further investigation, as the adaptive methods employed by PCa cells for survival under nutrient deficient conditions could be exploited for preferential therapeutic targeting of aggressive PCa cells [195, 196]. However, the feasibility of targeting such pathways and/or enzymes therapeutically will be dependent on whether healthy cells can tolerate such an intervention—many normal cells with highly proliferative activity such as immune cells and stem cells also reprogram their metabolism in a manner similar to cancer cells [186].

### Advancing PCa biomarker discovery

Urinary and serum-based gene signatures are being continually investigated to improve on those, which are currently available. Micro RNAs (miRNAs) have been shown to be involved in PCa development and progression and are appealing as biomarkers as they are seemingly stable under harsh conditions and detectable in both urine and serum [197, 198]. miR-155 has been shown to be over-expressed in a number of cancers, including PCa, and it has been found that combination of serum miR-155 and PSA measurements are diagnostic of PCa at an early stage, and also reflective of the clinicopathological features of PCa [198]. Jeon et al. have reported on a panel of 7 miRNAs, which has shown potential as a biomarker for PCa tumour grade [197]. Connell et al. have recently described gene-based Prostate Urine Risk (PUR) signatures, which can be used to classify PCa based on the D’Amico risk types. This versatile urine biomarker system is based on the RNA expression of 36 gene probes, including PCA3, TMPRSS2-ERG and HOXC6, and has been proposed as a tool for predicting the need for therapeutic intervention for men on AS [199]. While the pleothora of new gene-based PCa tests is promising for management of PCa, they are most applicable for early stage PCa as a means of ruling out aggressive disease. The intermediate PCa setting remains challenging for accurate prognosis, as current tests do not direcly detect tumour aggressiveness [200]. Tools to support the idenficiation of prostate tumours that will ultimately evade therapy or acquire treatment resistance, in advance of histopathological signs of tumour aggression, are actively being investigated [200]. Germ-line mutations in the BRCA2 gene have been linked with more aggressive PCa and resistance to ADT [201]. This has inspired further research into prognostic germline loci as minimally invasive biomarkers for stratification of indolent versus aggressive PCa. Germline variants are not unique to the tumour. However, focusing on loci that are specifically associated with methylation in the tumour has revealed that germline genotypes can modulate the PCa tumour epigenome, contributing to the development of aggressive PCa [202]. Zhao et al. have recently developed a urinary DNA methylation biomarker-based assay—ProCUrE—consisting of 6 genes; APC, GSTP1, HOXD3, KLK10, TBX15 and TGFβ2. The ProCUrE assay has demonstrated utility in predicting clinically significant PCa [203]. Circulating tumour cells (CTCs) can also be detected in the blood and are appealing as biomarkers due to the fact that they directly reflect the molecular expression profile of the tumour itself. Analysis of RNA extracted from CTCs led to the identification of a 12-gene panel which, in combination with PSA, achieved an AUC of 0.927 for prediction of clinically significant PCa [204]. While reports such as this are encouraging, the practicalities if CTC isolation present some limitations for routine sampling and biomarker analysis.

With the goal of understanding the genetic basis of aggressive cancer and treatment resistance, the PCAWG consortium has been established. This technical working group was set up to consolidate raw sequencing data covering a range of tumour types. To date, whole-genome sequencing data has been collected from thousands of male and female samples across 38 tumour types. The ultimate goal of this consortium is to engage a genomics community that will include healthcare providers, pharmaceutical companies, data scientists and clinical trial groups to build a comprehensive knowledge resource [205]. In a similar vein, Gerhauser et al. have reported on the development of PRESCIENT—a knowledge-based framework for genomics-informed PCa patient stratification and therapeutic targeting. They have compiled a comprehensive molecular catalogue of early-onset PCa, which details the earliest somatic mutation events, as a means of monitoring the molecular evolution and clinical trajectories of PCa [206].

Although advances in genomics-based research are exciting, only 10% of variation in protein abundances are actually explained by changes in the transcriptome [207]. This is impotant as proteins are arguably the most important functional molecules in the cell and therefore the clinical potential of protein biomarkers is high—especially for routine monitoring—as their expression can reflect disease activity in real time [207].

### Application of proteomics for novel biomarker discovery and development

Proteomics has had a tangible impact on biomarker discovery in PCa. A useful cancer protein biomarker would be a protein measurable in body fluids or tissues that could reflect the presence of cancer and provide information on the cancer’s stage, aggressiveness and how well the patient is responding to therapy and likehood of recurrence [208]. It quickly becomes apparent that a single protein (such as PSA) is unlikely to fulfil criteria for a viable biomarker and that a combination of multiple protein biomarkers will provide greater utility for improved PCa diagnosis and monitoring [209]. According to Rifai et al. the process of identifying new protein biomarkers is undertaken in four main stages, beginning with an initial discovery phase and ending with a final evaluation phase [210]. This process requires technologies that will allow for fast and consistent identification of proteins spanning the expansive dynamic range of the disease proteome [211]. Proteomics-based biomarker discovery can be performed in a wide variety of biological sample types; however, when it comes to to identifying a clinically useful protein biomarker there are advantages and disadvantages associated with each biological sample type (Table 3).

**Table 3:** Sample selection for biomarker discovery

	Tissue		Body fluids		
	Biopsy	Needle biopsy	Serum and plasma	Urine	Prostatic fluid and seminal plasma
Advantages	Direct analysis of tumor protein expression/activation		Non-invasive collection	Non-invasive collection	Minimally invasive collection
	Diagnostic markers		Fast and low-cost sample preparation	High volume	Rich in prostate-derived proteins
	Prognostic markers		Diagnostic markers	Rich in prostate-derived proteins	Fast and low-cost sample preparation
	Most useful for patient stratification in terms of response to therapy		Prognostic markers	Fast and low-cost sample preparation	Diagnostic markers
				Diagnostic markers	Prognostic markers
				Prognostic markers	
Limitations	Invasive collection		Low abundance of potential biomarkers	Low abundance of potential biomarkers	Low abundance of potential biomarkers
	Limited quantity		Dynamic concentration range	Dynamic concentration range	Dynamic concentration range
	Must be snap-frozen within 30 min from collection		Intra and inter-patient variability in composition	Intra and inter-patient variability in composition	Intra and inter-patient variability in composition
	Complicated sample preparation Tissue Sampling Errors			Variability in sample collection	

Table adapted from Pin et al. [209]

### Antibody-based proteomics

#### Enzyme-linked immunoabsorbant assay (ELISA)

Clinical evaluation of novel disease biomarkers was previously reliant on immunoassays due to their proposed specificity for the target analyte, sensitivity, and high throughput [212]. For a long time Enzyme Linked Immunoabsorbant Assay (ELISA) was the gold standard for protein detection in patient serum samples. In a typical double antibody sandwich ELISA, an antibody attached to the bottom of a well provides both antigen capture and immune specificity while another antibody linked to an enzyme provides the detection and amplification factors for protein detection [213]. As multiplexed protein measurement has become of increasing interest, the ELISA technique has been modified to allow for multiplexed measurement of protein biomarkers in a 96-well plate format. Many studies aimed towards the evaluation of potential PCa biomarkers have availed of this technique, however, a wide variety of variable factors are known to affect the performance characteristics of an ELISA. These include; the antibodies used, the temperature, the pH and the antibody incubation time [212, 214]. The most significant limitation to this technique is that antibodies do not yet exist for all proteins in the human proteome, which thereby rules out ELISA as a strategy for evaluating many novel protein biomarkers [215].

### Protein microarrays

Protein microarrays can also be used for protein profiling in serum samples. With this technique, thousands of proteins are printed and immobilized onto a glass slide, which allows for the simultaneous analysis of serum proteins in a high throughput fashion [216]. This technique is not extensively used as a means of evaluating PCa biomarkers. One group did report on its application for studying the expression of a HERV-KGAG protein in relation to the clinical progression of PCa. Here it was shown that there was an increased frequency of autoantibodies for HERV-KGAG protein in patients with advanced PCa, making it one of the first retroviral cancer antigens reported in humans [217]. However, similar to ELISAs, protein microarrays are expensive and also rely on the availability of antibodies. As interest in the development of highly specific and high throughput techniques for biomarker evaluation increases, moving away from traditional antibody-based techniques and branching out into nanotechnology offers a broad spectrum of innovative methods to meet the associated requirements for biomarker discovery and validation [216].

### Aptamer-based assays

Immunosensors based on aptamer interactions are becoming a favorable approach for sensitive detection of low molecular weight analytes of interest. Aptamers are DNA or RNA molecules with tridimensional conformation that gives them high affinity for specified biomolecules of interest [218]. In contrast to antibodies, aptamers can be easily modified, are smaller in size, cheaper to produce and can be generated against a wide variety of different target molecules [219]. Most aptamers are directly selected against the target analyte and are considered to be more sensitive than an antibody for the same analyte. Problems of capture-reagent cross reactivity and non-specific adsorption to surfaces are greatly reduced with aptamer-based platforms [220]. Aptamer technology has been successfully applied for the detection of PSA in both PCa cell biopsies and human serum. With aptamer-based technology, PSA is detectable at levels as low as the fg/ml range, with high specificity [221]. A modification of this platform is the SOMAscan assay, which uses slow off-rate modified aptamers (SOMAmers). These are single stranded DNA aptamers that contain pyrimidine residues carrying hydrophobic entities at their 5′ position. The affinity of SOMAmers is considerably higher than that of simple RNA or DNA aptamers [222]. Moreover, the platform is highly automated and scalable to allow for high sample throughput [223]. This technology is therefore considered an ideal platform for protein biomarker discovery and evaluation as it has the capacity to detect in excess of 1125 proteins in a single analysis, using minimal amounts (< 100 μl) of serum [224, 225]. In a study by Mehan et al. the SOMAmer platform was used to quantify 1033 proteins simultaneously with sub-pM limits of detection and inter-assay CV of < 5% in human serum samples. This analysis resulted in a 7-marker signature for detection of lung cancer in current and former smokers with an AUC of 0.85 for all and 0.93 for squamous cell carcinoma [226]. This study therefore indicates the potential benefits of applying this technology for PCa-related biomarker research.

### Mass spectrometry-based proteomics for biomarker discovery

**Fig. 6:**
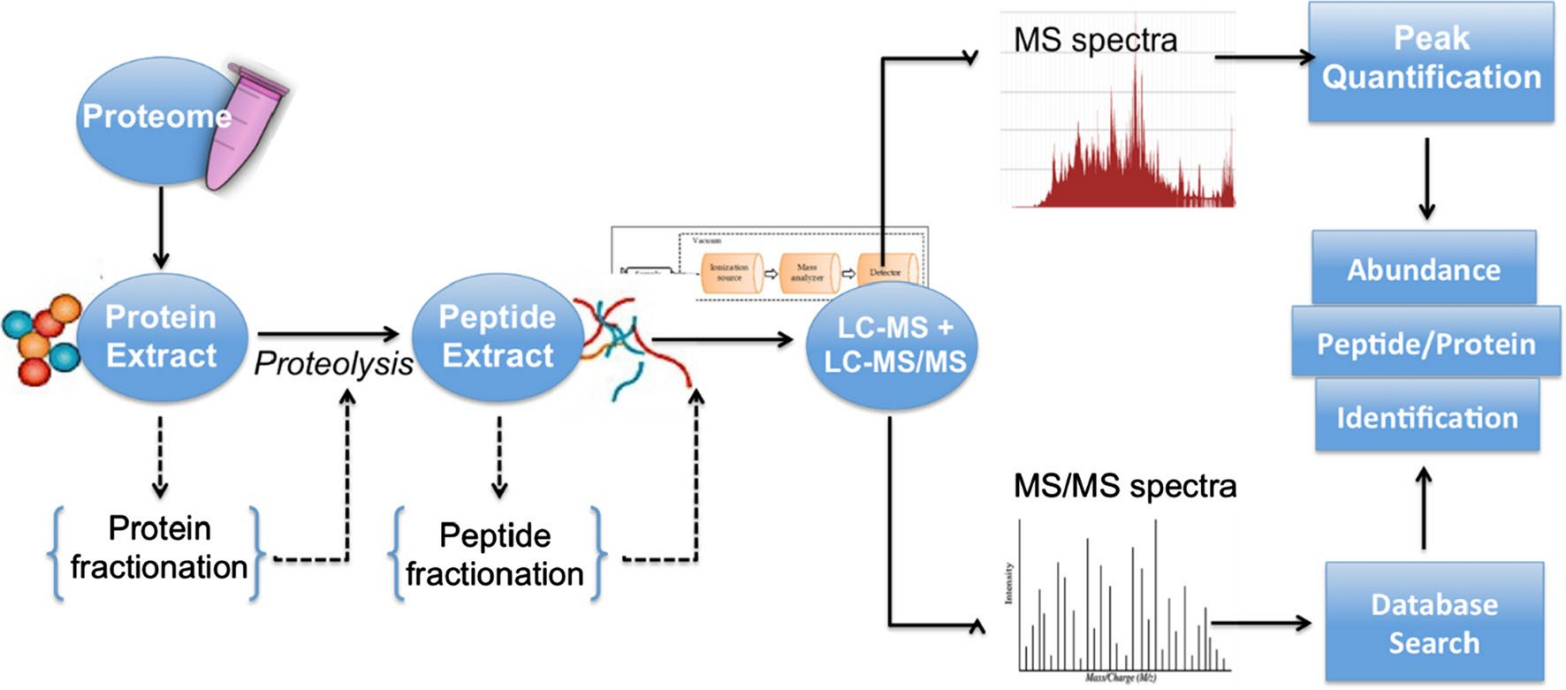
**Shotgun Proteomics Workflow.** The above schematic outlines the typical workflow implemented for shotgun proteomics. The core steps for sample preparation include protein extraction and proteolytic digestion of protein material into peptides. Optional steps include protein/peptide fractionation for increased proteome coverage of complex samples. Peak quantification and database searching are the key bioinformatics steps required for peptide/protein identification and label-free quantification

Over the last number of years mass spectrometry has emerged as an invaluable technology for the quantification of thousands of proteins as well as their modifications, localization, turnover and interaction partners [214]. It has been reported that over 70% of known proteins have been identified through mass spectrometry-based discovery experiments [227]. The global analysis of complex protein samples is often referred to as ‘shotgun’ proteomics. The workflow involved for such analysis follows three main experimental steps: (i) protein extraction, (ii) enzymatic digestion and peptide separation and (iii) peptide/protein identification and quantification (Fig. 6). The recent literature has many examples of mass spectrometry being applied for the identification of PCa biomarkers in clinical samples [228–230] (Table 4). It has also been applied for deciphering disease mechanisms—such as development of radio-resistance and response to therapy—through analysis of ex vivo disease models [231, 232]. For the purposes of biomarker discovery, ‘hybrid’ instruments are widely used due to their unparalleled analytical specificity. Hybrid mass spectrometers typically refer to high-resolution instruments that are coupled to a front-end component that enables fragmentation of peptides (Q-ToF, Triple-TOF, Q-Orbitrap). These analyzers can now fragment several thousand peptides per hour [233]. Thus emphasis is now shifting towards ‘deeper’ mass spectromtrey-based discovery experiments to detect the remaining 30–35% of uncharacterized proteins [234]. To this end, one of the afore-mentioned hybrid instruments, the Triple-TOF, has enabled a new peptide detection strategy called “Sequential Windowed Acquisition of all THeoretical ions” (SWATH). SWATH continuously fragments all peptides within stepped mass-to-charge ratio (m/z) windows [235]. The SWATH method relies on generation of peptide spectral libraries and produces permanent MS/MS maps of all analytes, that are above the detection limit of the respective instrument, in a biological sample [236–238]. A one-off SWATH analysis can provide a comprehensive dataset that can be used to answer various clinical/biological questions, making it a desirable technological tool in large cancer research facilities and consortiums. As this technology continues to develop, it is anticipated that there will soon be MS spectral libraries to represent peptides covering the entirety of the human proteome. Latonen et al. have recently applied SWATH-MS for an integrative characterization of PCa, with comprehensive proteomic analysis of BPH, untreated PCa and CRPC. This has led to the identification of several molecular and pathway events that had not previously been identified from transcriptomic studies [239]. Importantly, SWATH has proven capable of achieving identification of novel disease relevant cancer biomarkers in plasma [240, 241]. This is impressive considering that a common limitation with regard to biomarker identification in blood is that a large number of candidates are generally categorized as general inflammaltory response proteins, or proteins involved in lipid transport and coagulation, which are not specific to any one disease type [240]. Limited sample availability, which is generally a caveat in PCa biomarker research, has also been addressed. Guo et al. have developed an MS method, which optimizes sample preparation and mass spectrometric and computational elements, to facilitate highly reproducible and accurate quantification of thousands of proteins from biopsy-scale tissue samples at high throughput [242]. This optimized method combines pressure cycling technology (PCT)-with standard SWATH and has been applied for investigatons of FFPE. Most human tissue specimens are archived as FFPE blocks, however, there has long been a concern around the protein quality of FFPE samples due to formalin-induced chemical modifications to protein structure [243]. Zhu et al. have applied PCT-SWATH to the analysis of 224 PCa FFPE and corresponding fresh frozen (FF) tissue samples that had been stored for up to 8 years. Herein the authors reported enhanced biomarker discovery from FFPE in comparison to the FF counterpart [243]. Protein and mRNA degredation is a concern for any type of clinical sample. Hence, Shao et al. have sought to develop a scoring system for monitoring the degree of protein degredation—the Proteome Integrity Number (PIN)—in clinical samples. When applied to a clinical cohort they have shown that, although detectable, protein degredation has minimal impact on proteomic measurements and is independent of mRNA degredation [244]. Authors were also able to establish PIN as an accurate indicator of sample quality. This is important for proteomic studies of large multi-site clinical cohorts, where pre-analytical sample variailty cannot be precisely controlled [244]. SWATH technology is continuing to evolve with a view to becoming a platform that is compatible with requirements for routine analysis of clinical samples. For example, Sun et al. have reported on a microflow, single-shot, short gradient SWATH MS method for accelerated biomarker discovery and verification. This accelerated method can quantify 80% of detectable proteins using just 17% of the standard instrument time [245]. Network-based methods for studying MS-based proteomic data have been advocated as a more reliable means for biomarker discovery, with the premise that if co-ordinated overexpression of groups of proteins within a cluster is observed, with the exception of one, it is likely that that one protein is a biologoical outlier or a ‘false negative’. These network-based data analysis algorithms have been shown to be robust against noise and missing data and are thus considered superior to traditional analytical strategies [246]. Examples of some promising PCa biomarkers that have been identified using mass-spectrometry platforms, and their clinical application are summarised in Table 4.

**Table 4:** Examples of potential PCa biomarkers discovered in clinical samples by mass spectrometry

Biomarker	Sample type	Clinical application	MS technique	Publication year	Refs
16 Protein panel	Serum	Prediction of PCa recurrence	Discovery by label-free LC–MS/MS; Validation by MRM-MS	2013	Morrissey et al. [278]
Cholesterol, dihydrosphingomyelin, phophatidylcholine, egg phosphatidylcholine and egg phosphatidylethanolamine	Serum	Detection of PCa	ESI–MS/MS	2014	Patel et al. [279]
Ureidoisobutyric acid, Indoylacroylglycine, N-acetylvanilalinine	Urine	Detection of PCa	LC-HRMS	2014	Goto et al. [280]
41 Protein panel	Serum	Prediction of PCa recurrence	Discovery by label-free LC–MS/MS; Validation by MRM-MS	2015	Tonry et al. [281]
3 Protein panel	Urine	Detection of PCa	LC–MS/MS	2015	Overbye et al. [282]
Inter-alpha-trypsin inhibitor heavy chain H2, CD44 antigen, Immunoglobulin gamma 2 heavy chain, Cadherin-13	Serum	Aggressive v non aggressive PCa	PRM-MS	2015	Thomas et al. [283]
PAP and Galectin-3	Urine	Detection of PCa	Discovery by label-free LC–MS/MS; Validation by MRM-MS	2015	Geisler et al. [284]
136 Protein panel	Urine	Early diagnosis of PCa	MRM-MS	2015	Percy et al. [285]
Sphingosine	Tissue	Differentiation of PCa from BPH	LC–MS (metabolomics)	2016	Ren et al. [286]
3 Protein panel	Serum	Detection of PCa	Discovery by iTRAQ 3D LC–MS; Validation by ELISA	2016	Larkin et al. [287]
Lactoferrin	Tear	Differentiation of PCa from BPH	MRM-MS	2016	You et al. [288]
Claudin 3	Plasma (exosomes)	Differentiation of PCa from BPH	LC–MS discovery, ELISA validation	2017	Worst et al. [289]
Free amino acids: methionine, 3-methylhistidine, serine, sarcosine and proline	Serum + urine	Detection of PCa	LC–ESI–MS/MS	2017	Derezinski et al. [290]
12 protein panel	Urine	Early diagnosis of PCa	MRM-MS	2017	Shi et al. [230]
Furan, p-xylene	Urine	Detection of PCa	GC–MS	2018	Jiminez-Pacheco et al. [291]
56 N-glycopeptide panel	Urine	Differentiation of PCa from BPH	Discovery by label-free LC–MS/MS; Validation by PRM-MS	2018	Kawahara et al. [292]
3 Protein panel	Serum	Prediction of survival in metastatic PCa	2DE-MS	2018	Cho et al. [293]
Sarcosine and related metabolites	Urine	Early diagnosis of PCa	MRM-MS	2018	Yamkamon et al. [294]
Ferritin (heavy and light chain)	Urine	PCa Diagnosis	2DE-MS	2019	Zhao et al. [295]
Morse et al. multivariate Metabolomic classifier	Tissue		DESI-MSI	2019	Morse et al. [296]
Panel of 4 heavy metals	Serum	Prediction of PCa risk	Inductively coupled plasma mass spectrometry (ICP-MS)	2019	Lim et al. [297]
4 protein panel	Tissue	Distinguish between low and high grade PCa	Discovery by label-free LC–MS/MS; Validation by PRM-MS	2019	Kawahara et al. [298]
20-Metabolite panel	Serum	Prediction of PCa recurrence	NMR + LC–MS	2019	Clendinen et al. [299]
Phospho-lipid panel	Tissue	Prediction of PCa aggression	MALDI-MSI	2019	Randall et al. [300]
75 Protein panel	FFPE	Detection of PCa and differentiation of PCa from BPH	DIA-MS + verification by MRM-MS	2020	Sun et al. [245]

PCa: prostate cancer; LC–MS/MS: liquid chromatography mass spectrometry; ESI–MS: electrospray ionisation mass spectrometry; LC-HRMS: liquid chromatography high resolution mass spectrometry; MRM-MS: multiple reaction monitoring mass spectrometry; PRM-MS: parallel reaction monitoring mass spectrometry; GC–MS: gas chromatography mass spectrometry; 2DE-MS: 2 dimensional gel electrophoresis mass spectrometry; DESI-MSI: Desorption electrospray ionisation–mass spectrometry imaging; NMR: nuclear magnetic resonance; MALDI-MSI: matrix-assisted laser desorption ionization mass spectrometry imaging; DIA-MS: data independent acquisition mass spectrometry

### Mass spectrometry-based proteomics for biomarker development

For the purposes of evaluating the role of identified proteins as potential biomarkers, a targeted proteomic approach provides excellent sensitivity for the detection of proteins in biological samples [247]. Selected reaction monitoring (SRM)—otherwise known as multiple reaction monitoring (MRM)—enables high throughput, cost-effective assay development for quantification of selected proteins of interest. Targeted MRM assays are considered the mass spectrometry equivalent to a Western blot or ELISA. However, proteins are identified through the detection of specified combinations of precursor and product ion m/z’s of preselected proteotypic peptides—thereby eliminating the need for antibodies [248]. MRM enables quantification of hundreds of proteins simultaneously at low limits of detection with high accuracy. Moreover, MRM-triple quadrupole mass spectrometers also have a wide dynamic range which makes them ideal for analysis of protein expression in serum or plasma—arguably the biological fluid of choice for a clinical test [249, 250]. A similar technique, parallel reaction monitoring (PRM), has also been introduced to further improve on accuracy and selectivity for quantification of lower abundant peptides [251]. The PRM process is very similar to MRM, although the instrumentation is different; PRM is performed on a hybrid Quadrupole-Orbitrap as opposed to a triple quadrupole mass spectrometer and there is no requirement to pre-select product ions [252, 253]. PRM is reported to offer greater sensitivity and overcomes some of the limitations of MRM with regard to filtering out interfering signal from complex biological samples. However, in the field of PCa research, PRM has been more widely applied for exploratory experiments or wide screen analyses of samples [254, 255]. An overview of the proteomic technologies applied for biomarker discovery and development is provided in Table 5. For ultimate clinical application, MRM assays have been further progressed.

**Table 5:** Overview of proteomic platforms used for biomarker development

Category	Platform	Multiplex capability	Application	LOD	Advantages	Limitations
Antibody-Based	ELISA	96 proteins per assay	Biomarker evaluation & validation	pg/mL	Highly sensitive for protein(s) of interest	Dependant on antibody availability Influenced by non-standardised variables e.g. temperature, pH, antibodes used Large amounts of protein lysate required
	Protein microarrays	> 1000 proteins per screen	Biomarker evaluation & validation Biomarker discovery	pg/mL	High throughput for multiplexed analysis	Reliant on availability of antibodies Expensive Biased to pre-selected proteins if used for biomarker discovery Two antibodies required per protein
	Proximity extension assay (PEA)	Up to 100 proteins per array	Biomarker discovery	pg/mL	High throughput Highly sensitive for proteins of interest	Expensive Biased to pre-selected proteins if used for biomarker discovery Two antibodies required per protein
Aptamer-based	Somascan	Up to 2000 proteins per screen	Biomarker evaluation & validation Biomarker discovery	fg/mL	Aptamers cheaper to produce More sensitive than antibody-based techniques Aptamers available for wider range of molecules High throughput Minimal sample required	Biased to pre-selected proteins if used for biomarker discovery
Mass Spectrometry-based	DDA e.g. LC–MS/MS	1000′s of proteins	Biomarker discovery	ng/mL	High throughput Minimal sample required Unbiased screen of all detectable proteins No requirement for antibodies or somamers	Complex sample preparation required Enrichment techniques required to detect very low abundant proteins in clinical samples
	DIA e.g. SWATH & DIA PASEF	1000′s of proteins	Biomarker discovery	ng/mL	High throughput Minimal sample required Unbiased screen of all detectable proteins No requirement for antibodies or somamers Increased coverage of sample proteome Has been optimised for analysis of complex sample types such as FFPE tissue	Complex sample preparation required Enrichment techniques required to detect very low abundant proteins in clinical samples Requires specialised Mass Spectrometer Data storage
	MRM	Up to 100 proteins per run	Biomarker evaluation & validation	ng/mL	Highly selective for proteins of interest High throughput No requirement for antibodies/somamers Wide dynamic range	Complex sample preparation required Can be affected by interfering signal from complex biological samples Labour intensive method development
	PRM	> 100 proteins per run	Biomarker evaluation & validation Biomarker discovery	ng/mL	Greater sensitivity than MRM Interfering signals filtered out Method development less labour intensive than MRM	Complex sample preparation required Measurements not as precise as MRM Higher LOQ than MRM

LOD: Limits of detection; DDA: Data dependent acquisition; DIA: Data independent acquisition; SWATH:; PASEF:Sequential Window Acquisition of All Theoretical Mass Spectra; Parallel accumulation—serial fragmentation; MRM: multiple reaction monitoring; PRM: parallel reaction monitoring

Huttenhain et al. recently developed a repository of MRM assays for over 1000 previously identified cancer-associated biomarkers. This study also demonstrated the applicability of MRM assays to reproducibly and accurately quantify biomarker candidates across a large number of patient samples [256]. As such, multiplexed MRM technology is considered to have the greatest potential to bridge the gap between compiling panels of biomarker candidates and proving their clinical utility in patients [237, 257]. Indeed, MRM is already routinely used in a clinical setting and various CLIA-approved MRM-based assays are now available as diagnostic tests [258]. One of the most established of these is the LC–MS/MS measurement of 25-hydroxy metabolites of vitamin D2 and vitamin D3. This assay is now in routine diagnostic use and the automated LC–MS/MS system allows up to 180 tests to be performed in a 24 h period [259, 260]. Mass spectrometry coupled to immunoaffinity separations has also been applied to establish an MS-based clinical assay for measurement of variants of a protein biomarker for renal failure—cystatin C [261]. A similar assay has been established for measurement of beta-2-glycoprotein in plasma samples. As well as being an FDA approved biomarker for active rheumatoid arthritis and kidney disease, this protein has also been heavily associated with PCa progression [262]. A number of MS-based assays are also now offered for the detection of insulin resistance and type-2 diabetes through measurement of retinol binding protein [263], insulin-like growth factor I and II [264] and insulin [265, 266]. Two commercially available MS-based assays have been developed for improved management of lung cancer—Veristat and Express Lung. The Veristat assay is an imaging-MS based algorithm that measures 8 distinct m/z features and has been validated as a clinically useful serum protein test [267–269]. The Express Lung test is an MRM-based assay measuring 5 diagnostic and 6 normalisation proteins. It has been validated as a proteomic classifier for identification of benign lung nodules with a high negative predictive value [270]. Nuclea Biotechnologies also offer LC–MS/MS-based tests to measure serum levels of c-peptide, proinsulin, apoplipoprotein A1 and Apolipoprotein B. Although these commercial tests are not yet FDA approved, they are currently catagorised as lab-developed tests (LDTs) and have been developed and characterized under CLIA requirements. To support development of MS technology in the clinical field, a number of consortia have been established to instill guidelines for robust experimental design and measures to reduce false discovery in biomarker development. Examples include the NCI CPTAC and the Early Detection Research Network (EDRN). The most important criteria that must be met for validation of novel biomarkers is outlined in Table 6. Further details on the various proteomic platforms used for biomarker development and their key features, can be found in Table 5, as well as in reviews published previously [271].

**Table 6:** Recommended analytical criteria for biomarker development

Validation factor	Definition [277]
Accuracy	Level of agreement between biomarker and standard measure of disease presence/outcome
Bias	Repeated biomarker measurements do not result in under/overestimation of disease activity
Repeatability	Precision of biomarker under standard "unchanged" conditions e.g. between batches
Intermediate precision	Precision of biomarker test in spite of laboratory variations e.g. time analysed, operator, equipment, calibration
Reproducibility	Precision of biomarker test between laboratories with potential variability in operators and measuring systems
LoD	The smallest amount of analyte that can be detected with a specified probability
LoQ	The smallest amount of analyte that can be quantitatively determined with acceptable precision
Linearity	Measured quantity values are directly proportional to the amount of biomarker in the experimental matrix

Table adapted from Yee et al. [277]

## Conclusions

Although the mortality rate for patients diagnosed with PCa is relatively low, there is a critical need for more appropriate clinical management of the disease to ensure that patient’s quality of life is preserved as much as possible throughout the duration of the disease. Importantly, there is also a pertinent need to be able to predict PCa recurrence and detect CRPC at an earlier stage. PSA, while still the ‘gold-standard’ biomarker for informing on disease progression, is not sufficiently specific as it is also elevated in non-cancerous prostate diseases. It is now widely accepted that panels of biomarkers that can be measured in a multiplexed fashion, are of greater clinical utility than measurement of a single molecular marker. This is reflected in the biomarker studies summarised in Table 4, which cover a range of clinical applications for management of PCa. Increasing knowledge of the prostate tumour microenvironment as well as advancements in multiplexed proteomic technologies will be of significant advantage to these efforts. For example, Intratumoral heterogeneity (ITH) is a defining characteristic of PCa and it is important to recognize how this influences the utility of protein biomarkers [246, 272]. Ultimately, the true clinical role of any identified biomarker test will require rigorous assessment to ensure that it is (i) cost-effective, (ii) can provide additional information to what is already provided by PSA and (iii) can be easily incorporated into routine workflows in clinical laboratories—this following robust technical validation according to criteria outlined in Table 6. Ideally, biomarker tests will be measureable in patient blood or urine samples as this will allow for routine, non-invasive monitoring of disease progression throughout the (often-times) lengthly duration of this disease. A range of databases for storage of proteomic datasets are now available for storage of mass spectrometry-derived datasets, which can be accessed freely and re-analysed by researchers. These are extremely useful within the field of biomarker research for in silico validation of biomarker signatures [196, 273]. Zhong et al. have also compiled a comprehensive imaging resource—curating a collection of PCa microscopy imaging data from hundreds of prostate specimans—to complement high throughput proteomics and genomics data. Therefore, there now exist a large number of shared data resources with excellent potential for reuse in both biomedical and computational studies [274]. Hence any experiment which characterises the molecular composition of clinical samples is highly valuable for identification of clinically applicable and functionally relevant biomarkers for PCa.

## Data Availability

Not applicable.

## Abbreviations

PSA: Prostate Specific Antigen
PCa: Prostate Cancer
BPH: Benign Prostate Hyperplasia
CP: Chronic Prostatitis
FDA: Food and Drug Administration
ERSPC: European Randomised Study of Screening for Prostate Cancer
PLCO: Prostate, Lung, Colorectal and Ovarian Cancer Screening Trial
USPSTF: US Preventative Services Task Force
EUA: European Urology Association
STHLM3: Stockholm 3 Study
fPSA: Free PSA
hK2: Hexokinase 2
MSMB: Microeminoprotein beta
MIC1: Macrophage Inhibitory Cytokine 1
DRE: Digital Rectal Exam
TRUS: Transrectal ultrasound
MRI: Magnetic resonance imaging
ESUR: European Society of Urogenital Radiology
GS: Gleason Score
RP: Radical Prostatectomy
IDC-P: Intraductal carcinoma of the prostate
CR: Cribiform carcinoma
TNM: Tumour node metastasis
AS: Active surveillance
ADT: Androgen deprivation therapy
AUA: American Urology Association
CHRT: Combined hormone and radiation therapy
EBRT: External beam radiation therapy
SBRT: Stereotactic body radiation therapy
HDR: High dose radiation
LDR: Low dose radiation
IGRT: Image guided radiation therapy
IMRT: Intensity modulation radiation therapy
SRT: Salvage radiation therapy
PAP: Prostatic acid phosphatase
GM-CSF: Granulocyte–macrophage colony stimulating factor
CAR: Chimeric antigen receptor
PSMA: Prostate specific membrane antigen
PSAV: PSA velocity
PSADT: PSA doubling time
P2PSA: [-2] Proenzyme PSA
Phi: Prostate health index
PTEN: Phosphatase and tensin homologue
CE-IVD: Commission for in vitro diagnostics
FFPE: Fresh frozen paraffin embedded
RT-PCR: Real time polymerase chain reaction
PCA3: Prostate cancer antigen 3
CLIA: Clinical laboratory improvement ammendments
LDT: Laboratory developed test
mCRPC: Metastatic castrate resistant prostate cancer
DHT: Dihydrotestosterone
AR: Androgen receptor
AR-V: Androgen receptor variants
HIF: Hypoxia inhibitory factor
CA-IX: Carbonic anhydrase IX
miRNA: MicroRNA
PUR: Prostate Urine Risk
CTC: Circulating tumour cell
ELISA: Enzyme linked immunoabsorbant assay
MS: Mass Spectrometry
SWATH: Sequential Windowed Acquisition of all THeoretical Ions
PCT: Pressure cycling technology
PIN: Protein integrity number
SRM: Selected reaction monitoring
MRM: Multiple reaction monitoring
PRM: Parallel reaction monitoring
LC–MS/MS: Liquid chromatography tandem mass spectrometry
EDRN: Early detection research network
ITH: Intratumoural heterogeneity
